# Interaction and UV-Stability of Various Organic Capping Agents on the Surface of Anatase Nanoparticles

**DOI:** 10.3390/ma7042890

**Published:** 2014-04-10

**Authors:** Mohsin Raza, Angelika Bachinger, Nina Zahn, Guido Kickelbick

**Affiliations:** 1 Vienna University of Technology, Institute of Materials Chemistry, Getreidemarkt 9/16, Vienna 1060, Austria; E-Mails: mohsin_qau5@yahoo.com (M.R.); angelika.bachinger@swerea.se (A.B.); 2 Saarland University, Inorganic Solid State Chemistry, Am Markt Zeile 3, Saarbrücken 66125, Germany; E-Mail: n.zahn@mx.uni-saarland.de; 3 Current address: Swerea SICOMP AB, Argongatan 30, Mölndal 43122, Sweden

**Keywords:** titania, surface functionalization, nanoparticles, photocatalytic decomposition

## Abstract

Anatase nanoparticles synthesized by the sol-gel method were surface-functionalized with long alkyl chain coupling agents as compatibilizers for a nonpolar environment, containing different anchor groups for surface interaction namely phosphonate (dodecyl phosphonate), carboxylate (dodecanoic acid), sulfate (sodium dodecyl sulphate), and amine (dodecyl amine). It was shown that the surface of the nanoparticles can be functionalized with the various surface groups applying similar reaction conditions. The kind of surface interaction was analyzed applying FTIR spectroscopy. The phosphonate and the carboxylate groups interact with the surface via quite strong covalent or coordinative interactions, respectively. The sulfate and amine based coupling agents on the other hand exhibit electrostatic interactions. UV stability studies of the surface bound groups revealed different degradation mechanisms for the various functionalities and moreover showed that phosphonates are the most stable among the investigated surface capping groups.

## 1. Introduction

Semiconductor photocatalysis represents a versatile tool for the removal of pollutants from water and air and has thus gained considerable interest in the field of Green Chemistry in recent decades [1–4]. Titanium dioxide nanoparticles have been applied extensively for this purpose, because their band gap energy and the redox potentials of the photo-generated electrons and holes allow the degradation of organic pollutants upon illumination with solar light. Anatase is generally accepted to exhibit highest photocatalytic activity among the crystalline phases of titanium dioxide [4,5]. However, an enhancing effect of both, rutile and brookite on the photocatalytic activity of anatase has been reported [6,7].

The modification of metal oxide nanoparticles with organic ligands, coupling agents and surfactants is also a hot topic these days [8]. A large variety of investigations have been conducted in the past regarding the adsorption of surfactants to metal oxides for specific interaction with molecules or materials, to prevent agglomeration or to induce self-organization [9]. Organic compounds, which are majorly used as metal oxide modifiers, are carboxylic acids, thiols, silanes, phosphonates, and rarely amines [8,10–12]. There are many discussions about the mechanism of interaction of ionic surfactants to the metal oxide nanoparticles. Some proposed models suggest the presence of electrostatic interaction, covalent bonding, formation of bilayers and micelles at higher concentrations [13,14]. The factors that are important in surface-functionalization of metal oxides with the above mentioned organic compounds are pH conditions and surface properties of the metal oxides.

It is known that the anatase surface exhibits amphoteric properties [15–17]. The adsorption of acidic compounds has been proven to take place via acid-base reaction on Brønsted basic sites. The adsorption of basic compounds on the other hand does not take place on Brønsted acidic sites, but rather on Lewis acidic sites [16,18]. However, Brønsted acidity has often been created on titanium dioxide surfaces by introduction of nitrates or sulfates [18,19].

In the first part of the present work, different functionalities were attached to the surface of anatase nanoparticles, taking advantage of the acidic as well as the basic sites. It is known from literature that phosphonic acid (DPA) and dodecanoic acid (DDA) usually interact by formation of strong chemical bonds with the TiO_2_ surface [20,21]. As far as we know no systematic studies of dodecylsulphate (SDS) and amines were carried out and compared to the bonding scheme of the previously mentioned groups.

The different interactions of the organic molecules with the surface of the titania nanoparticles might lead to unequal photocatalytic properties. The question that arises is whether the photocatalytic activity can even be stopped by a high surface coverage because a bare TiO_2_ surface is usually necessary for the oxidation and reduction processes. Previous studies on the photocatalytic decomposition of phosphonates, carboxylic acids, sulfates and amines were conducted with regard to environmental applications and hence the preclusion of the release of toxic intermediates was aimed [22–27]. Numerous investigations focused on the so-called Photo-Kolbe reaction, the decarboxylation of carboxylic acids over TiO_2_ for the production of alkanes [28]. However, only few publications exist where the degradation was studied with regard to the anchoring of these species to the titanium dioxide surface [14,29]. Experiments on the stability of phenyl phosphonate on titanium dioxide under photocatalytic conditions by Vioux and coworkers indicated the degradation of organic species and the formation of a phosphate species [14]. Franch *et al.* [29] studied the degradation of maleic acid with regard to its adsorption on the TiO_2_ surface. They found a complete disappearance of the carboxylate vibrational signals indicating that the bonding between the carboxylate group and the TiO_2_ surface is instable.

The goal of the present study was the attachment of various coupling agents containing different anchor groups but the same organic alkyl chains to the surface of anatase nanoparticles under the identical reaction conditions. The interaction of the organic groups with the inorganic surface was systematically studied by FT-IR spectroscopy. Subsequent investigations involved studies on the photocatalytic stability of the different capping molecules. The effect of different kinds of chemical interactions of the chemisorbed molecules on their photocatalytic decomposition was also investigated in the study.

## 2. Results and Discussion

**Figure 1.**
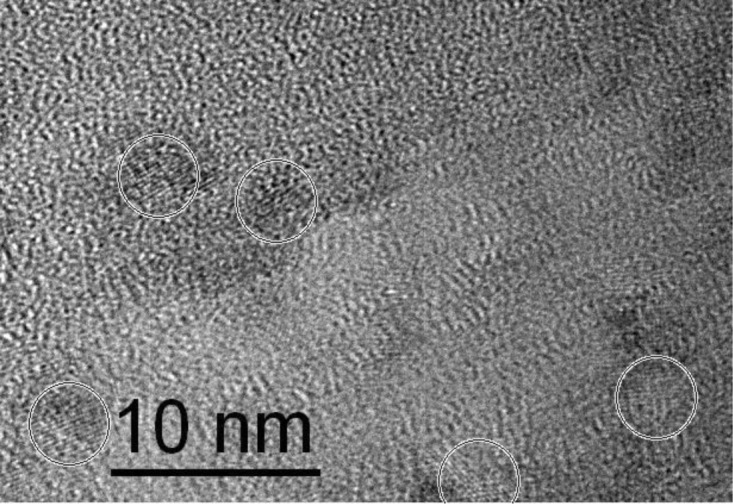
TEM micrograph of titania nanoparticles.

Titania nanoparticles were prepared by the hydrolysis and condensation of titanium isopropoxide under highly acidic conditions achieved by addition of nitric acid according to Choi *et al.* [30]. Particle size of the titania nanoparticles was investigated using dynamic light scattering (DLS) from the dried sample redispersed in water. DLS analysis revealed particle diameters of 5 ± 0.2 nm (supporting information, Figure S1). These results were confirmed by HR-TEM shown in Figure 1 where crystalline particles of ~5 nm are circled.

Surface properties of the particles were investigated by FT-IR analysis (Figure S2). Intense vibrational signals at 1380 and 1620 cm^−1^ indicated the presence of nitrate and water on the surface, which is in agreement with literature reports [30,31].

XRD patterns of titania nanoparticles (Figure S3) revealed pure anatase phase (2001-JCPDS file number 21-1272) exhibiting a crystallite size of 3.9 nm. Nitrogen sorption experiments revealed a surface area of 144 m^2^/g for the prepared titanium dioxide nanoparticles.

In a subsequent step, titania nanoparticles were functionalized with sodium dodecyl sulfate (SDS), dodecanoic acid (DDA), dodecyl amine (DDAmine) and dodecylphosphonic acid (DPA). The modification reactions were carried out by stirring at room temperature in an ethanol/water mixture containing the respective capping agent. The experiments were conducted in acidic pH conditions keeping in view the investigations of Dunphy-Guzman *et al.* [32] which proved that suspensions of titanium dioxide particles in water are more stable in acidic conditions than in basic conditions owing to the tendency of titania nanoparticles to carry positive surface charge. Nitrates present on surface might not be involved in the surface functionalization of acidic surfactants like carboxylates, phosphonates and sulfates but they can play a role in the surface functionalization of titania with basic surfactants like amines. In case of SDS the concentration was kept under its critical micelles concentration, *i.e.*, 8.12 × 10^−3^ M to make all molecules available for surface functionalization [33].

The influence of the pH and the concentration of capping molecules on the surface coverage and bonding properties were investigated and the photocatalytic stability of the modified particles was tested. The bonding of the anchoring molecules to the titanium dioxide surface was investigated by infrared spectroscopy. Thermogravimetric analysis and elemental analysis were applied to study the surface coverage.

**Figure 2.**
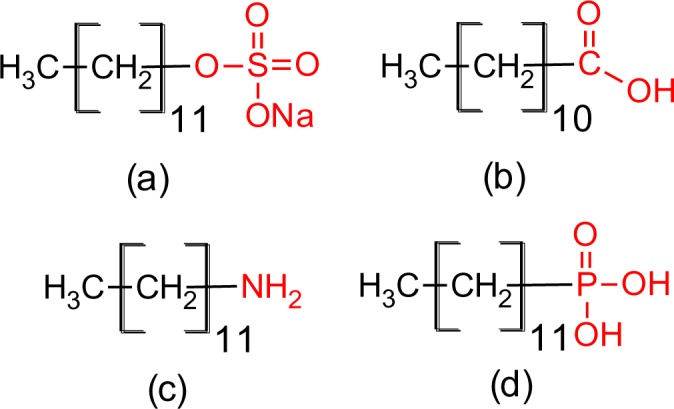
Four surface capping molecules used for titania (**a**) sodium dodecyl sulfate; (**b**) dodecanoic acid; (**c**) dodecyl amine; (**d**) dodecyl phosphonic acid.

The four coupling agents with four different functional groups used for modification of titania nanoparticles are presented in Figure 2.

### 2.1. Infrared Spectroscopy

#### 2.1.1. SDS@TiO_2_

**Figure 3.**
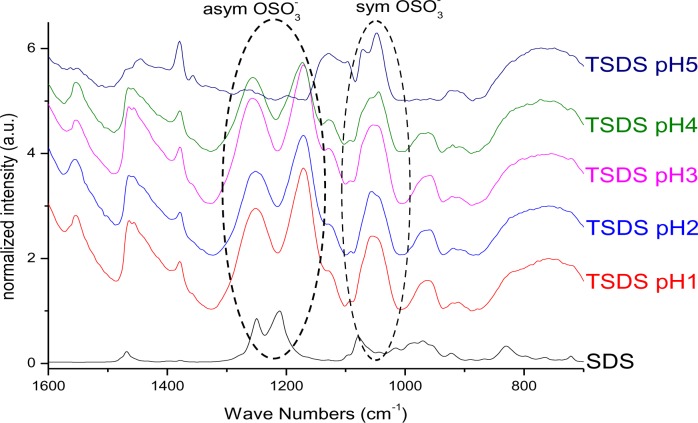
FT-IR (1600–700 cm^−1^) of SDS@TiO_2_ functionalized at pH 1, 2, 3, 4 and 5 in HNO_3_.

Changes in vibrational frequencies of the sulphate group have previously been used to determine its bonding mode [34]. For free sulphate only one signal for the S–O asymmetric stretching vibration can be observed at ~1100 cm^−1^. In outer-sphere complexes the band is shifted to higher wavenumbers owing to distortion and the symmetric stretching band at ~970 cm^−1^ becomes visible. For inner-sphere complexes the symmetry is lowered and the ν_as_ band splits into two or three signals between 1050 and 1250 cm^−1^ depending on the bonding mode. In monodentate complexes two signals are identified, while in bidentate complexes the band splits into three signals [35]. Literature reports on the interaction of sulphate species with titanium dioxide surfaces indicate bidentate coordination after heat treatment, while at tempartures below 200°C only electrostatic interaction has been found [36]. The infrared spectrum of SDS (Figure 3) exhibited a strong doublet between 1200 and 1300 cm^−1^, which can be assigned to the ν_as_ signal of S–O. The bands between 950 and 1100 cm^−1^ are ascribed to ν_s_ vibration [37]. Hence, no direct bond between the sulphate and the sodium exists. For particles modified with SDS, the ν_as_ vibrational signals were shifted to lower wavenumbers and the doublet was splitted into two signals (Figure 3). Hence, it is concluded that an inner sphere complex is formed upon modification at pH 1 and 2. However, the splitting of the doublet might also be assigned to the dissolution of Ti^4+^ at highly acidic conditions and the formation of bulk titanium sulphate on the surface of the particles. This result is in agreement with investigations by Kingman and coworkers [37], who obtained similar spectra upon drying of SDS films on hematite. They found that the ν_as_ band splitting increased upon drying and assigned this effect on the dissolution of Fe^3+^ ions which form bulk iron dodecylsulphate. They stated that this precipitate dominates the spectra but can be redissolved in water and hence, in aqueous environment the doublet exhibits again smaller splitting. In fact, it was found that upon vigorous washing of the particles in water, the splitting disappeared and a doublet was observed at 1240 and 1208 cm^−1^ which is in agreement with various literature reports on SDS on different surfaces [33,36–38]. Hence, no direct bonding of the sulphate to the titanium dioxide surface exists, but SDS is rather adsorbed by electrostatic interactions. At pH 5 the signals are much weaker indicating a lower surface coverage which is in good agreement to the TGA measurements. The use of hydrochloric acid does not change the degree of modification or the resulting binding modes (Figures S5 and S29).

**Figure 4.**
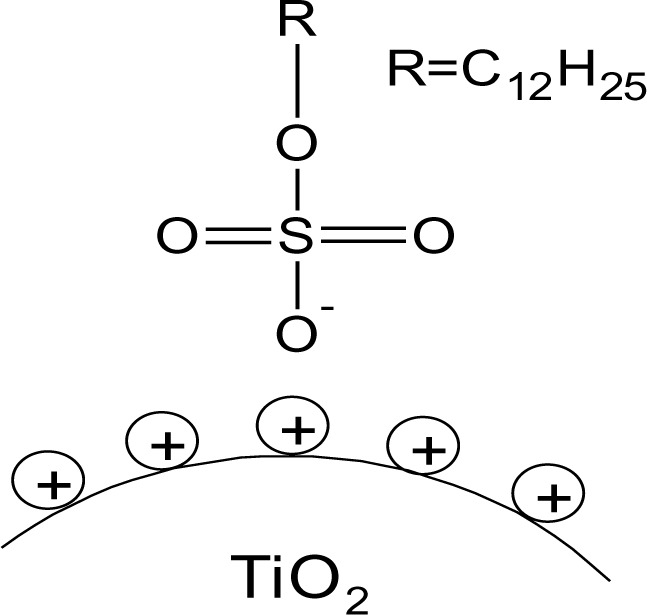
Schematic representation of SDS@TiO_2_.

On the bases of these evidences the proposed model of SDS@TiO_2_ nanoparticles is an electrostatic interaction between the negatively charged sulfate and the positively charged titania surface (Figure 4), which is destroyed at higher pH values.

#### 2.1.2. DDA@TiO_2_

**Figure 5.**
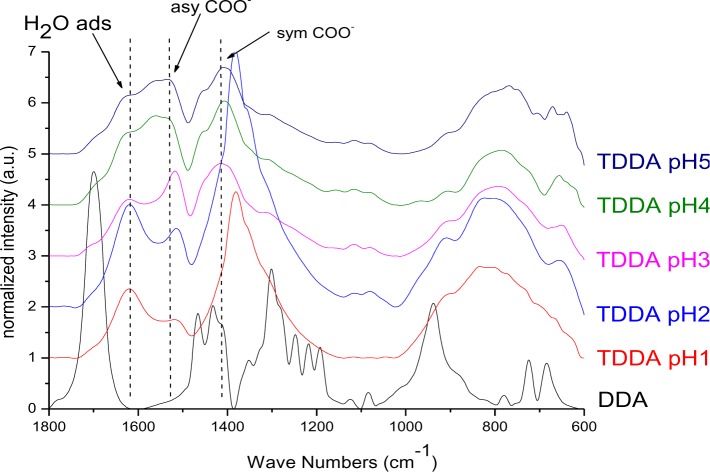
FT-IR spectra (1800–600 cm^−1^) of DDA@TiO_2_ at pH 1, 2, 3, 4 and 5.

**Figure 6.**
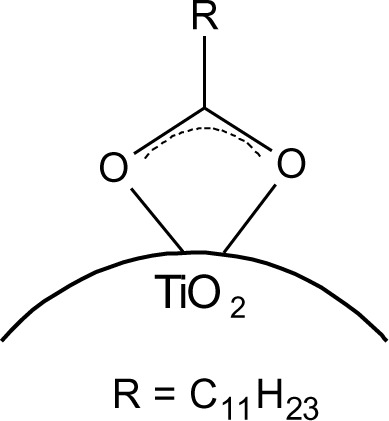
Schematic representation of DDA@TiO_2_.

The particles modified with DDA were characterized regarding the bonding mode of DDA by infrared spectroscopy. The FT-IR spectra of DDA@TiO_2_ (Figure 5) revealed a disappearance of the C=O vibration at 1700 cm^−1^ compared to the spectrum of DDA, while two new signals at 1519 and 1412 cm^−1^ were identified. These vibrations are assigned to symmetric and asymmetric stretching vibrations of COO^−^. It is concluded that DDA is bound to the titania surface as a carboxylate species and the free acid has been successfully removed by washing. Moreover, according to previous studies on carboxylates, four possible types of interaction exist: monodentate, bridging bidentate, chelating bidentate and ionic interaction. The type of interaction between the carboxylate and the metal oxide surface can be analyzed by the wavenumber separation of the asymmetric and symmetric COO^−^ vibrations [13,39,40]. Largest separation is obtained for monodentate interaction [13,41]. Literature reports on the assignment of medium and small separation regions however, are not unambiguous. Palacios and others claim that the wavenumber separation increases with increasing covalent character of the bond, owing to increasing asymmetricity [41,42]. Hence they assign medium separation to bidentate interaction and the smallest separation to ionic interaction [43]. Moreover, they state that the asymmetric vibrational signal is more intense than the symmetrical one for coordination compounds in contrast to ionic interactions. According to them, the distinction between chelating and bridging bidentate bonds is difficult, because the bond orders should not be different. However, it has been stated that the wavenumber separation should be smaller for chelating than for bridging compounds [44]. Other groups including Dobson and McQuillan stated that medium separation can be assigned to bidentate bridging or ionic interaction (150–180 cm^−1^) and the smallest separation is assigned to bidentate chelating interaction (60–110 cm^−1^) [13,40]. The separation in the prepared samples was 107 cm^−1^, which is assigned to a bidentate coordination bond in agreement with both theories. The wavenumber separation was in the lower range for bidentate bonds, indicating predominantly chelating interaction according to Dobson and others (Figure 6) [13]. This result is in agreement with earlier investigations by Wang and coworkers on the bonding of DDA on anatase nanoparticles [45]. However, it is not possible to gain information from the relative intensities of the signals, because both vibrations are overlapping with other signals (hydrogen bonded water at 1620 cm^−1^ and nitrate at 1380 cm^−1^). The shifts of the symmetric and asymmetric COO^−^ vibration were the same for all pH values, which means that there is no pH dependent change of the carboxylate binding mode. FT-IR spectra of DDA@TiO_2_ in range of 3050 to 2800 cm^−1^ revealed the presence of the characteristic alkyl signals (supporting information).

## 2.1.3. DDAmine@TiO_2_

**Figure 7.**
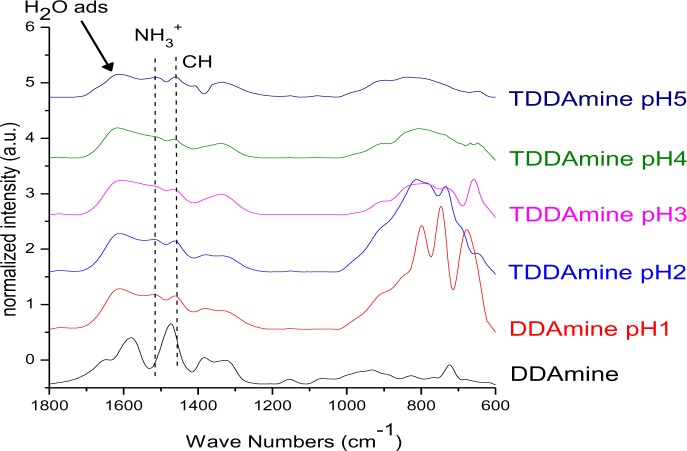
FT-IR spectra of DDAmine@TiO_2_ at pH 1, 2, 3, 4, and 5.

**Figure 8.**
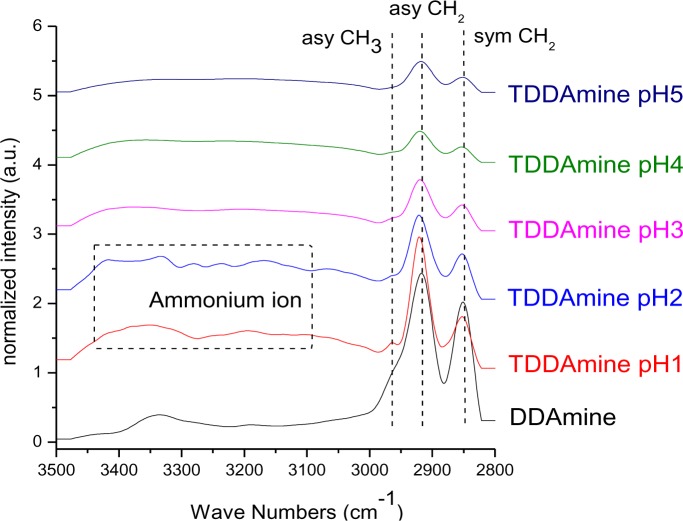
FT-IR spectra of DDAmine@TiO_2_ at pH 1, 2, 3, 4 and 5.

**Figure 9.**
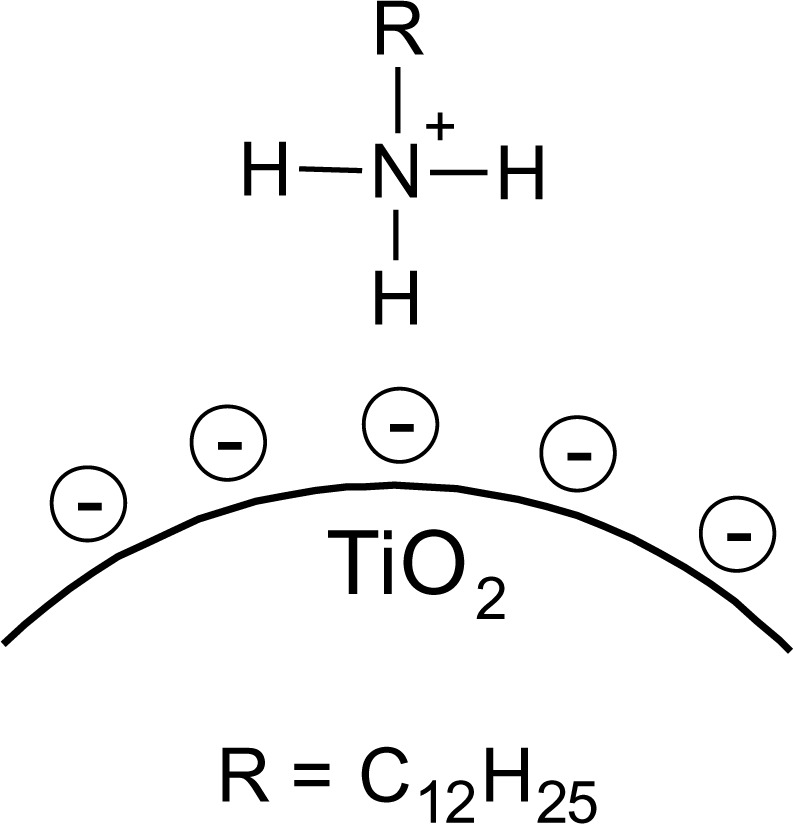
Schematic representation of bonding of DDAmine@TiO_2_.

It is known that amines can adsorb to titania surfaces at Lewis-acid sites, via hydrogen-bonds, or electrostatic interaction [15,16]. The latter is more likely to occur on anatase surfaces than on rutile, due to a higher proton acidity of the surface OH– groups [15,17]. Brønsted acid sites have been shown to be not involved in the bonding of amines to titanium dioxide, as no ammonium vibrations were identified. However, the treatment with nitrate or sulfate induces the formation of Brønsted acid sites which subsequently undergo acid-base reaction with amines [18]. Owing to the use of HNO_3_ in the synthesis of the anatase nanoparticles in this work, the surface is nitrated. FT-IR spectra of DDAmine@TiO_2_ (Figures 7 and 8) revealed an intense band at 1610 cm^−1^ which is assigned to the bending modes of adsorbed water. The N–H stretching vibration is overwhelmed by this signal in the spectra for particles modified with DDAmine. The intense signal at 1466 cm^−1^ and two weak vibrations at 1378 and 1305 cm^−1^ can be assigned to C–H vibrational signals (scissor, CH_2_ and CH_3_ deformation vibrations). For the particles modified with DDAmine, a signal at 1520 cm^−1^ is arising, which can be assigned to the NH_3_^+^ bending vibration [46]. While the NH_2_ stretching signal is observed at 3374 cm^−1^ in the spectrum of DDAmine, the modified particles exhibit various broad signals between 3100 and 3500 cm^−1^ indicating the presence of ammonium species and the formation of hydrogen bonds [47]. Hence, electrostatic interaction takes place with Brønsted acid sites induced by the nitrates present on the particles’ surface. The formation of ammonium species and the ionic interaction detected by infrared spectroscopy is in agreement with the results obtained by Ramis and Forzatti [18] (Figure 9). At higher pH values, the ammonium signals disappear, indicating interaction of the amine with Lewis acid sites. However, the absence of amine signals indicates that the disappearance of ammonium signals might rather be attributed to a lower surface coverage.

### 2.1.4. DPA@TiO_2_

**Figure 10.**
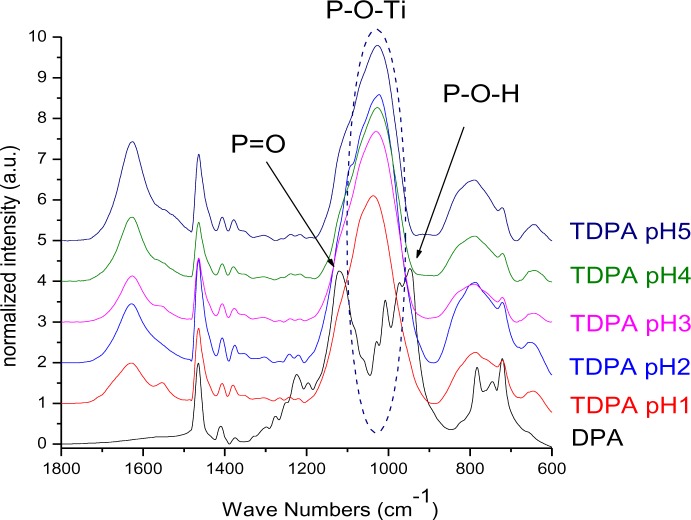
FT-IR spectra of DPA@TiO_2_ at pH 1, 2, 3, 4 and 5.

**Figure 11.**
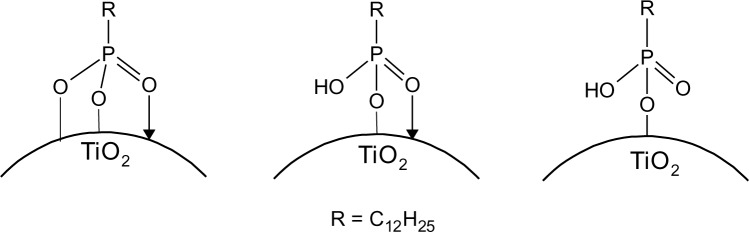
Schematic representation of DPA@TiO_2_.

The interaction of the phosphonate group with TiO_2_ was characterized by infrared spectroscopy. The heterocondensation of P–OH groups with surface hydroxyl groups and the coordination of phosphoryl oxygen to titanium lead to the formation of covalent bonds. The connection can be mono-, bi- or trivalent and also hydrogen bonds have been shown to be involved [21,48]. FT-IR spectra of DPA@TiO_2_ modified at pH 1, 2, 3, 4 and 5 (Figure 10) show strong bands for the coupling agents which is due to the high specific surface area of the particles in combination with the relatively high surface activity concerning attachment reactions resulting in a high degree of functionalization (see also surface coverage calculations in SI). The spectra reveal the disappearance of the P=O vibrational signal at 1220 cm^−1^ and the P–O–H signal at 950 cm^−1^. Moreover, a broad band was identified between 970 and 1180 cm^−1^, which can be assigned to P–O–Ti bonds [14]. These results are in good agreement with literature reports on the modification of titania with organic phosphonates and prove the covalent connection of DPA to the titanium dioxide surface [10]. However, investigations on the bonding modes of such species are difficult. Due to the formation of hydrogen bonds, the disappearance of P–OH and P=O vibrational signals do not prove trivalent bonding. Furthermore, due to a variety of factors that influence the phosphorus shift, only limited conclusions about the bonding mode can be drawn from ^31^P MAS NMR. Mutin and coworkers conducted ^17^O MAS experiments in order to determine the bonding mode of organophosphonates to titanium dioxide surfaces [21]. They observed chemical shifts consistent with bridging P–O–Ti bonds. Moreover, they concluded from the presence of residual P=O and P–OH bonds that several different binding modes are co-existent (Figure 11). The predominant bonding mode depends strongly on the studied system.

Hence, the bonding mode cannot be identified by infrared spectroscopy for the studied particles. However, covalent connection could be proven by the characteristic P–O–Ti signal.

### 2.2. Thermogravimetric Analysis and Elemental Analysis

#### 2.2.1. Effect of pH on the Surface Coverage

Surface coverage of the titania nanoparticles with all coupling agents at pH 1, 2, 3, 4 and 5 was investigated by thermogravimetric and elemental analysis. Percentage surface coverage and number of molecules per square nanometer of titania were calculated from the thermogravimetric results and the surface area of the anatase nanoparticles obtained by nitrogen sorption measurements (supporting information).

Thermogravimetric analysis of SDS@TiO_2_ revealed two different onset temperatures. The first onset at 250°C is assigned to the loss of the organic part of the SDS, while the second one at 570°C can be ascribed to the loss of SO_2_ as determined from TG-MS analysis. Mass losses below 200°C are assigned to the desorption of volatiles and the condensation of surface hydroxyl groups. The mass loss between 200 and 700°C was applied for determining the surface coverage.

**Figure 12.**
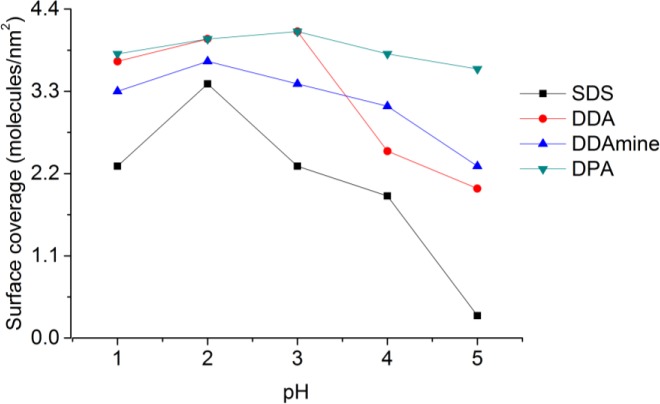
Effect of pH on surface coverage of titania by the four coupling agents.

At pH 2 the highest surface coverage was obtained for SDS as observed from Figure 12. The surface coverage decreased upon increasing pH, owing to a decreasing positive surface charge, which leads to weaker ionic interaction with negative sulfate ions. At pH 5 no organic species were detected on the particles’ surface, because it is close to the point of zero charge of titania (pH_PZC_ ~ 5.9 for TiO_2_) [49]. However, a lower surface coverage was also detected for particles modified at pH 1 compared to pH 2. This effect might be assigned to better association of protons to the sulfate at highly acidic conditions. Elemental analysis of the five samples of titania functionalized with SDS at different pH conditions confirmed the observations from TGA and maximum amount of sulfur was found at pH 2 and minimum for the sample at pH 5 (supporting information).

Thermogravimetric analysis of DDA@TiO_2_ revealed one onset temperature at 300°C, which is assigned to the oxidation of the alkyl chain. The surface coverage was calculated from the mass loss between 200 and 700°C.

Highest surface coverage for DDA could be achieved by modification at pH 3 (Figure 12). The surface coverage is decreasing at higher pH values, which is assigned to a decreasing positive surface charge and hence lower interaction with the dissociated carboxylic acid. At pH values below 3, however, the surface coverage is also decreasing. This effect might be attributed to worse dissociation of the acid at extremely low pH values. The influence of the pH on the surface coverage is much lower for DDA compared to SDS (Figure 12). This observation is attributed to a higher influence of the surface charge on the ionic interaction with SDS compared to the covalent interaction with DDA.

For DDAmine@TiO_2_ thermogravimetric analysis exhibited one onset temperature at 280°C. Owing to the weak electrostatic interaction detected for the amine, the mass loss might be ascribed to the desorption of DDAmine. However, the onset temperature is in the range of the alkyl chain oxidation temperature (compare with DDA and SDS). Hence, it is assumed that the alkyl chain is rather degraded than desorbed. The surface coverage was calculated from the mass loss between 200 and 700°C.

Highest surface coverage was detected for particles modified at pH 2 (Figure 12). The coverage is decreasing at lower pH owing to an increasing positive surface charge inhibiting the interaction with the basic compound. However, surface coverage is also decreasing when the pH is elevated above 2. This effect might be assigned to the removal of nitrate from the titanium dioxide surface and hence the decrease of Brønsted acid sites. Elemental analysis confirmed the results obtained by TGA and revealed maximum N content for particles modified at pH 2.

Thermogravimetric analysis of DPA@TiO_2_ revealed one onset temperature at 300°C, which can be assigned to the oxidation of the alkyl chain. The surface coverage calculated from the mass loss between 200 and 700°C was highest for particles modified at pH 3 (Figure 12). At higher pH values the surface coverage was decreasing, which is assigned to a lower positive surface charge leading to weaker interaction with the dissociated phosphonic acid. However, also at pH values below 3 the surface coverage decreased. This effect might be assigned to lower tendency for dissociation of the phosphonic acid at highly acidic conditions. Elemental analysis confirmed the results obtained by TGA and revealed the highest phosphorus content at pH 3.

#### 2.2.2. Effect of Capping Agent Concentration on the Surface Coverage

**Figure 13.**
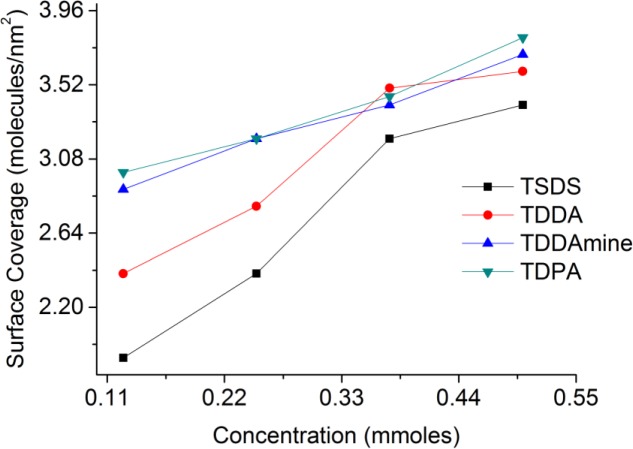
Effect of concentration on surface coverage of titania by the coupling molecules.

A set of experiments were conducted to functionalize titania nanoparticles with different concentrations of coupling molecules at the best suitable pH conditions for each molecule to investigate the effect of the concentration of capping agent on the surface coverage of titania nanoparticles. For SDS and DDAmine, these experiments were conducted at pH 2 whereas pH 3 was selected for DDA and DPA. Figure 13 shows the relation of surface coverage and concentration calculated with the help of surface area of the titania nanoparticles and thermogravimetric analysis of the surface-functionalized samples. It is clear that with the increase in concentration from 0.125 to 0.5 mmol of each coupling molecule, the surface coverage increases in the same order.

### 2.3. Stability of Capping Agents under Photocatalytic Conditions

**Figure 14.**
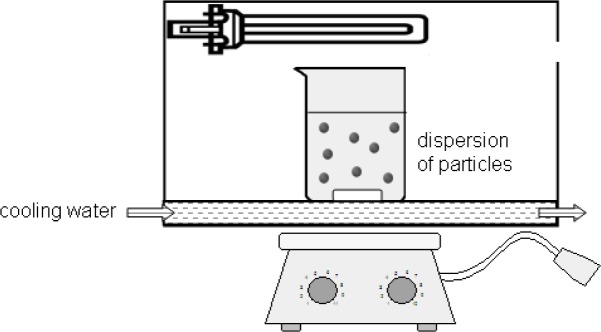
Experimental setup for the illumination experiments.

It is assumed that the different interactions of the coupling agents with the titania surface can lead to unequal photodegradation mechanisms and kinetics. For this reason, anatase nanoparticles modified with DPA, DDA, SDS and DDAmine were illuminated under the same conditions. 0.2 wt% dispersions of DPA@TiO_2_, DDA@TiO_2_, SDS@TiO_2_ and DDAmine@TiO_2_ in mixtures of ethanol and water (4:1) were prepared and illuminated for 2 days in a water-cooled chamber equipped with two 9 W UVA black light lamps (Sylvana UVA BLB Lynx) as presented in Figure 14. Samples were withdrawn after 2, 4, 8, 24 and 48 h to investigate the degradation of the surface groups. The particles were centrifuged, washed with ethanol and dried under reduced pressure before FT-IR characterization.

After the functionalization with the different coupling agents, the nitrate signal at 1303 cm^−1^ disappeared in all cases. Exemplarily we also modified TiO_2_ synthesized with HCl as acid for the weakest bonding surface groups, namely SDS and the FT-IR spectra after modification were identical (Figure S5). Therefore, an influence of nitrate ions in the photocatalytic process can be neglected. We also compared the functionalization of P25 with SDS, however here we observed a modification which is drastically reduced, most likely because of the inactive P25 surface [50].

The absorbance maximum at 2921 cm^−1^ (asymmetric aliphatic CH stretching vibration) can be taken as a measure for the amount of alkyl chains present on the particles’ surface. Thus, the absorbance intensity (*A*) change of this signal under UV illumination was tracked. Assuming 1st order kinetics the rate law is:

$$k[A]=-\frac{d[A]}{dt}$$ (1)

After integration the following equation is obtained:

$$kt=\ln(\frac{A_{0}}{A})$$ (2)

**Figure 15.**
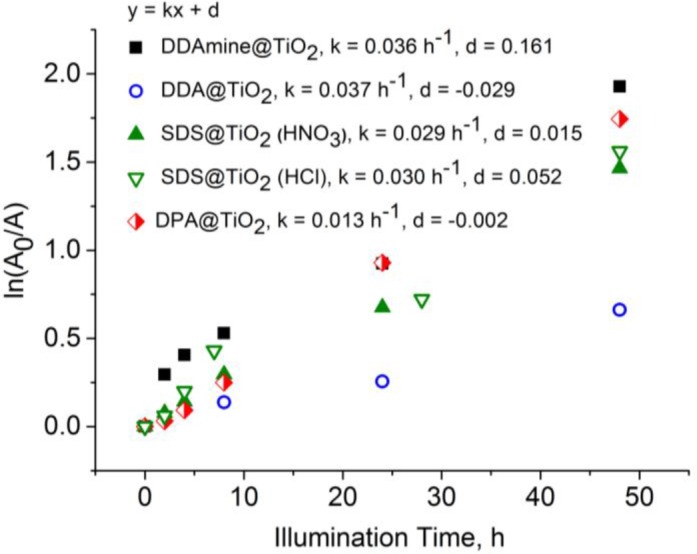
Absorbance at 2921 cm^−1^ of DPA @ TiO_2_, DDA @ TiO_2_, SDS @ TiO_2_ and DDAmine @ TiO_2_ after different times of illumination.

Thus, ln(*A*_0_/*A*) is plotted *versus* time to determine the reaction rate constant k. The kinetic plots and reaction rate constants obtained for the different capping agents are presented in Figure 15. The CH-signal decreased with increasing illumination time for all studied groups (detailed spectra in supporting information). It is concluded that the alkyl chain is degraded in all studied cases. However, as seen from the slopes in the graphs, the degradation proceeds much slower for DPA than it does in case of carboxylate, sulfate and amine.

No conclusions on the mechanism of degradation can be drawn from the CH stretching region. For this reason, the region between 700 and 1800 cm^−1^ was studied, where the characteristic vibrational signals of the studied head groups are located.

**Figure 16.**
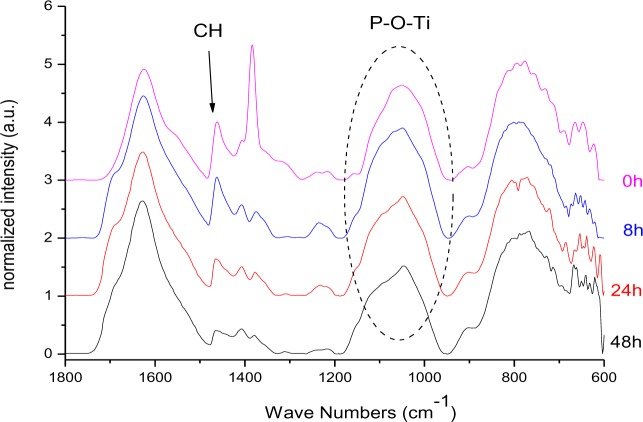
FT-IR spectra (KBr: 2mg sample, 150 mg KBr) of DPA @ TiO_2_ after different times of illumination.

In Figure 16 the FT-IR spectra of DPA@TiO_2_ after different illumination times normalized to the Ti–O–Ti band at 800 cm^−1^ are presented. The spectra revealed that the CH_3_ vibration at 1462 cm^−1^ (asymmetric CH deformation) was decreasing while the broad signal at 1050 cm^−1^, which is assigned to the P–O–Ti bond, did not lose intensity during illumination. Hence, it is concluded that the P–O–Ti bond was stable against UV-irradiation, while the alkyl chain was degraded. It is assumed that a phosphate species was left on the particles’ surface after illumination. In fact, after prolonged illumination for 16 days ^31^P MAS NMR investigations revealed the presence of a phosphate species (supporting information).

**Figure 17.**
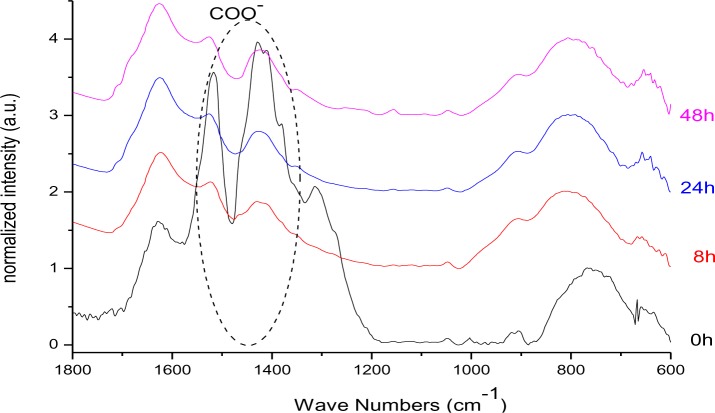
FT-IR spectra (KBr: 2 mg sample, 150 mg KBr) of DDA@TiO_2_ after different times of illumination.

From the FT-IR spectra of DDA@TiO_2_ (Figure 17) it can be concluded that the nitrate signal at 1384 cm^−1^ as well as the band at 1620 cm^−1^ (which is assigned to the bending vibrations of adsorbed water and thus increasing during illumination due to the photo-induced hydrophilicity effect [51]) were overlapping with the stretching modes of the carboxylate anion signals. Thus, an eventual change in intensity of the COO^−^ band cannot be definitely excluded. Nevertheless, it is assumed that the C–O–Ti interaction was stable against UV-irradiation, because no change in intensity was observed for the bands at 1412 and 1519 cm^−1^ after the interfering nitrate signal had disappeared (after 8 h). Furthermore, the bonding mode did not change as no shifting of the carboxylate bands was observed. Unfortunately, ^13^C CPMAS NMR could not confirm this assumption, because such species cannot be distinguished from the chemisorbed dodecanoic acid. The UV-stability of the C–O–Ti bond confirmed the assumption that the interaction is a chemical one. An electrostatic interaction between COO^−^ and a positively charged titania surface would be weakened upon illumination due to the extensive formation of radicals, which react with ions present on the surface of the particles. The fast degradation of DDA compared to DPA can be assigned to the effective charge transfer over the carboxy-group as found by Kaletas *et al.* [52]. However, another explanation could be the hindrance of the direct hole oxidation pathway by the presence of phosphate species as stated by Zhao and coworkers [53].

For SDS@TiO_2_ the degradation was measured observing the CH signal at 1425 cm^−1^ as well as the OSO_3_^−^ bands at 1248 and 1178 cm^−1^. The infrared spectra for various illumination times are presented in the supporting information (Figure S27). The CH signal was decreasing very fast upon illumination, while at the same time the OSO_3_^−^ bands disappeared. Thus, the radicals formed upon illumination reacted with the ionic species present on the titania surface and the electrostatic interaction was broken.

In case of DDAmine@TiO_2_ the illumination experiments were carried out with the sample modified at pH 1, which showed mainly electrostatic interaction. FT-IR spectra of DDAmine@TiO_2_ after different times of illumination (normalized to the Ti–O–Ti band at 800 cm^−1^) (see supporting information, Figure S28) revealed that the CH signal at 1460 cm^−1^ was decreasing as it did also for the above discussed coupling agents. At the same time the NH_3_^+^ vibration at 1510 cm^−1^ were decreasing [15,54]. Thus, it is concluded that the interaction between titania and amine was not stable upon UV illumination and the electrostatic interaction between NH_3_^+^ and the Ti–O groups was broken due to reaction of photogenerated radicals with the ionic species. Hence, the degradation of covalently bound coupling agents proceeds by sequential degradation of the alkyl chain while the covalent interactions between the TiO_2_ surface and phosphate or carboxylate are stable under UV illumination. On the other hand, coupling agents which exhibit electrostatic interaction with the anatase surface, are ‘desorbed’ from the TiO_2_ surface due to the formation of radicals which react with the ionic species present on the surface.

## 3. Experimental Section

### 3.1. Materials

Titanium isopropoxide (TIP), sodium dodecylsulphate (SDS), dodecanoic acid (DDA) and dodecylamine (DDAmine) were purchased from Sigma Aldrich and used as received. Dodecylphosphonic acid was prepared by a procedure according to Kosolapoff *et al.* [55,56]. Methanol and absolute ethanol were purchased from Acros and used as received. Nitric acid (65%) and NaOH were purchased from Merck.

### 3.2. Measurements

Infrared spectra were recorded on a Bruker Tensor 27 instrument (Bruker, Rheinstetten, Germany) either working in ATR Micro Focusing MVP-QL with a diamond crystal or in transmission mode using KBr pellets as a sample matrix. Spectra were recorded with 32 scans at a resolution of 4 cm^−1^. The software used for analysis was OPUS^™^ version 4.0. For kinetic studies, a baseline correction was carried out. Thermogravimetric analyses were performed on a Netzsch Iris TG 209 C (Netzsch Corp., Selb, Germany) with a 414 TASC controller in a platinum crucible with a heating rate of 10 K/min under synthetic air. High-resolution transmission electron microscopy (HR-TEM) micrographs were performed on a TECNAI F20 FEGTEM (Philips Electron Optics, Eindhoven, the Netherlands) at the University Services Centre for Transmission Electron Microscopy (USTEM), Vienna University of Technology. DLS measurements were performed with an ALV/CGS-3 compact goniometer system (ALV-GmbH, Langen, Germany) controlled by an ALV/LSE-5003 Multiple Tau Digital Correlator (ALV-GmbH) at a scattering angle of 90° and a temperature of 25°C. The light source was a Uniphase cylindrical 22-mW HeNe-laser (JDS Uniphase Corporation, Ottawa, ON, Canada) operating at a wavelength of 632.8 nm with vertically polarized light. Elemental analysis was carried out at the Microanalytical Laboratory at the University of Vienna. NMR spectra in solution were recorded on a Bruker Advance 300 instrument (^1^H: 250.13 MHz, ^13^C: 62.89 MHz, ^31^P: 101.25 MHz) equipped with a 5 mm broadband probe head and a z-gradient unit. Solid state NMR spectra were recorded on a Bruker AVANCE DPX300 equipped with a 4 mm broadband MAS probe head (^1^H: 299.87 MHz, ^13^C: 75.40 MHz, ^31^P: 121.39 MHz). Spectra were acquired using magic angle spinning (MAS) and high power proton decoupling at a rotor spinning rate of 4–9 kHz. Nitrogen sorption measurements were carried out on a Micrometric ASAP 2000 or ASAP 2010 instrument (Micrometric Instruments Co., Cleveland, OH, USA). The samples were degassed for 5 h at 40°C prior to measurement. For the interpretation of the data, the Brunauer, Emmett and Teller (BET) model was applied. XRD experiments of powders were carried out on a Philips X’Pert diffractometer (Philips, Eindhoven, the Netherlands, Cu Kα line: λ = 1.54060, 1.54439 Å) equipped with an XCelerator multi-channel detector, Bragg Brentano geometry and a silicon single crystal sample holder. The diffraction pattern was recorded between 5° and 90° (2θ) with 1 s/step and a step size of 0.02°.

### 3.3. Syntheses

#### 3.3.1. Synthesis of TiO_2_ Nanoparticles

Titania nanoparticles were synthesized by slight modification of a procedure by Choi *et al.* [32]. 500 mL of distilled water were placed in a 1000 mL round bottom flask and the pH was adjusted to 1.5 using nitric acid or hydrochloric acid, respectively. 2.5 mL (0.008 mmol) of Ti(O^i^Pr)_4_ were dissolved in 50 mL absolute ethanol in a dropping funnel and added drop-wise to acidified water under ice-cooling and vigorous stirring. The mixture was stirred overnight. The solvent was removed under reduced pressure at 40°C and the particles were dried under vacuum to collect a white powder. 0.6 g (94%) of titania nanoparticles were obtained.

DLS: 5 nm ± 0.2 nm. TEM: 5 nm. XRD: Anatase 100%, a (Å) = 3.79959, c (Å) = 9.4778; crystallite size: 3.9 nm. FT-IR (ν, cm^−1^): 3244, 1638, 1413, 1310, 795. TGA (mass loss, %): 30–400°C: 15.3%, 400–700°C: 0.5%. BET: 144 m^2^/g.

#### 3.3.2. Modification of TiO_2_ Nanoparticles with Sodium Dodecyl Sulfate (SDS)

0.1 g of titania nanoparticles were dispersed in 100 mL of distilled water in a 250 mL round bottom flask. 0.15 g (0.5 mmol) SDS were dissolved in a mixture of 5 mL water and 5 mL ethanol. This solution was added drop-wise to the dispersion of titania nanoparticles under vigorous stirring. The corresponding pH was adjusted using a 1 M solution of HNO_3_, HCl or NaOH, respectively. The system was stirred for two days and the particles were separated by centrifugation. The samples were washed repeatedly with water and ethanol and dried under reduced pressure at room temperature. The functionalization experiments were performed at pH 1, 2, 3, 4 and pH 5.

FTIR: 2951 cm^−1^ (ν_as_ −CH_3_); 2922 cm^−1^ (ν_as_ −CH_2_−); 2872 cm^−1^ (ν_s_ −CH_3_); 2852cm^−1^ (ν_s_ CH_2_−); 1248 cm^−1^ (ν_as_ OSO_3_^−^), 1178 cm^−1^ (ν_as_ OSO_3_^−^); 1064 cm^−1^ (ν_s_ OSO_3_^−^), below 800 cm^−1^ (ν Ti−O).

TGA (HNO_3_), mass loss (220°C to 650°C): pH 1: 15.00%; pH 2: 14.45%; pH 3: 14.05%; pH 4: 13.80%; pH 5: 8.58%.

TGA (HCl), mass loss (220°C to 650°C): pH 1: 15.95%; pH 2: 15.06%; pH 3: 14.15%; pH 4: 13.84%; pH 5: 8.60%.

To investigate the effect of concentration of SDS on the surface coverage, a set of experiments was conducted at pH 2, adjusted using a 1 M solution of HNO3. For 0.1 g of titania nanoparticles 0.13 g (0.5 mmol) of SDS were used for Sample-1, 0.1 g (0.375 mmol) for Sample-2, 0.067 g (0.25 mmol) for Sample-3 and 0.033 g (0.125mmoles) of SDS were used for Sample-4.

TGA, mass losses (220°C to 650°C): Sample-1: 21%; Sample-2: 20%; Sample-3: 15%; Sample-4: 12%.

#### 3.3.3. Modification of TiO_2_ Nanoparticles with Dodecanoic Acid (DDA)

0.1 g of titanium dioxide nanoparticles were dispersed in 30 mL of water in a 100 mL round bottom flask. 0.1 g (0.5 mmol) of DDA were dissolved in 5 mL of ethanol prior to its drop-wise addition in titania suspension. The functionalization experiments were performed at pH 1, 2, 3, 4 and 5. The pH was adjusted using a 1 M solution of HNO_3_ or NaOH.

The suspension was treated in an ultrasonic bath for one hour and stirred at room temperature for two days. The particles were separated by centrifugation, washed several times with ethanol and water and dried under reduced pressure at room temperature.

FTIR: 2951 cm^−1^ (ν_as_ −CH_3_); 2922 cm^−1^ (ν_as_ −CH_2_−); 2872 cm^−1^ (ν_s_ −CH_3_); 2852cm^−1^ (ν_s_ −CH_2_−); 1521 cm^−1^ (ν_as_ COO); 1412 cm^−1^ (ν_s_ COO); below 800 cm^−1^ (ν Ti−O).

TGA, mass loss (220°C to 550°C): pH 1: 19%; pH 2: 20%; pH 3: 21%; pH 4: 12%; pH 5: 11%.

Four samples of titania functionalized with different amounts of DDA were prepared at pH 3 to investigate the effect of concentration of DDA on the surface coverage. For each experiment, 0.1 g of titania nanoparticles were dispersed in 30 mL of water. 0.1 g (0.50 mmol) of DDA was used for Sample-1; 0.075 g (0.375 mmol) for Sample-2; 0.05 g (0.25 mmol) for Sample-3 and 0.025 g (0.125 mmol) of DDA were used for Sample-4.

TGA, mass loss (220°C to 550°C): Sample-1: 19%; Sample-2: 18%; Sample-3: 14%; Sample-4: 12%.

#### 3.3.4. Modification of TiO_2_ Nanoparticles with Dodecyl Amine (DDAmine)

0.1 g of titania nanoparticles were dispersed in 30 mL deionized water in a 100 mL round bottom flask. 0.093 g (0.5 mmol) of DDAmine were dissolved in 5 mL of ethanol and added drop-wise to the titania suspension. The functionalization experiments were performed at pH 1, 2, 3, 4 and 5. The pH was adjusted using 1 M solution of HNO_3_ or NaOH. The suspension was treated in an ultrasonic bath for one hour and subsequently stirred at room temperature for two days. The particles were separated by centrifugation and washed repeatedly with ethanol and dichloromethane. The samples were dried under vacuum overnight at room temperature.

FTIR: 2951 cm^−1^ (ν_as_ −CH_3_); 2922 cm^−1^ (ν_as_ −CH_2_−); 2872 cm^−1^ (ν_s_ −CH_3_); 2852cm^−1^ (ν_s_ CH_2_−); 1610 cm^−1^ (H_2_O); 1510 cm^−1^ (ν_as_NH_2_); 1600 cm^−1^ (ν_s_NH_2_); below 800 cm^−1^ (ν Ti−O).

TGA, mass loss (220°C to 550°C): pH 1: 15%; pH 2: 16%; pH 3: 15%; pH 4: 14%; pH 5: 10%.

Elemental Analysis: pH 1: 11.10% C, 2.5% H, 0.95% N; pH 2: 12.13% C, 2.91% H, 1.18% N; pH 3: 11.52% C, 2.32% H, 0.89% N; pH 4: 10.6% C, 2.40% H, 1.09% N; pH 5: 7.33% C, 1.81% H, 0.81% N.

Four samples of anatase nanoparticles functionalized with different amounts of DDAmine were prepared at pH 2 to investigate the effect of concentration of DDAmine on the surface coverage. For each experiment, 0.1 g of titania nanoparticles were dispersed in 30 mL of water. 0.093 g (0.50 mmol) of DDAmine were used for Sample-1; 0.070 g (0.375 mmol) for Sample-2; 0.046 g (0.25 mmol) for Sample-3 and 0.023 g (0.125 mmol) of DDAmine were used for Sample-4.

TGA, mass loss (220°C to 550°C): Sample-1: 16%; Sample-2: 15%; Sample-3: 14%; Sample-4: 13%.

#### 3.3.5. Modification of TiO_2_ Nanoparticles with Dodecylphosphonic Acid (DPA)

0.1 g of titania nanoparticles were suspended in 30 mL water in a single neck round bottom flask. 0.125 g (0.5 mmol) of DPA were dissolved in 5 mL of ethanol and added drop-wise to the suspension. The functionalization experiments were performed at pH 1, 2, 3, 4 and pH 5. The pH was adjusted using 1 M solution of HNO_3_ or NaOH. The particles were separated by centrifugation and washed repeatedly with distilled water and methanol. The samples were dried under reduced pressure at room temperature.

FTIR: 2951 cm^−1^(ν_as_ −CH_3_); 2922 cm^−1^ (ν_as_ −CH_2_−); 2872 cm^−1^ (ν_s_ −CH_3_); 2852cm^−1^ (ν_s_ CH_2_−); 1200 to 1000 cm^−1^ (ν_as_ P−O−Ti).

TGA, mass loss (220°C to 650°C): pH 1: 16%; pH 2: 16%; pH 3: 17%; pH 4: 15%; pH 5: 15%.

Elemental Analysis: pH 1: 9.28% C, 2.5% H, 0.85% P; pH 2: 11.13% C, 2.85% H, 1.03% P; pH 3: 11.58% C, 2.91% H, 1.18% P; pH 4: 9.6% C, 2.32% H, 1.08% P; pH 5: 8.33% C, 2.01% H, 0.9% P.

Four samples of titania functionalized with different amounts of DPA were prepared at pH 3 to investigate the effect of concentration of DPA on the surface coverage. For each experiment, 0.1 g of titania nanoparticles were dispersed in 30 mL water. 0.125 g (0.50 mmol) of DPA was used for Sample-1; 0.093 g (0.375 mmol) for Sample-2; 0.0625 g (0.25 mmol) for Sample-3 and 0.312 g (0.125 mmol) of DPA was used for Sample-4.

TGA, mass loss (220°C to 650°C): Sample-1: 23%; Sample-2: 21%; Sample-3: 17%; Sample-4: 15%.

#### 3.3.6. Stability of the Capping Agents under Photocatalytic Conditions

0.2 wt% dispersions of DPA@TiO_2_, DDA@TiO_2_, SDS@TiO_2_ and DDAmine @ TiO_2_ in mixtures of ethanol and water (4:1) were prepared and illuminated for 2 days in a water-cooled chamber equipped with two 9 W UVA black light lamps (Sylvana UVA BLB Lynx). Samples were withdrawn after 2, 4, 8, 24 and 48 h. The particles were centrifuged, washed with ethanol and dried under reduced pressure. The samples were characterized by KBr-IR. 2 mg ± 0.03 of the samples were homogenized and pressed with 150 mg ± 2 of KBr.

## 4. Conclusions

Anatase titania nanoparticles with a diameter of around 5 nm were functionalized with SDS, DDA, DDAmine and DPA in acidic pH conditions. It was confirmed that SDS and DDAmine interact electrostatically with the surface of titania whereas DDA bonds covalently to the titania surface through bidentate chelating mode and DPA bond covalently to the titania in a tridentate fashion. It has been shown that an optimal pH range exists for the modification with all studied capping molecules. The existence of such optimum is assigned to the dependence of the surface charge of the particles on the pH. For the interaction with acidic and anionic species, a highly positive surface charge is favorable. For interaction with the basic amine, the presence of nitrates on the particles’ surface, which is true at moderately low pH, increases the interaction. At highly acidic conditions, however, the acidic compounds exhibit lower tendency for dissociation, leading to inhibition of the interaction with the particles. For amines on the other hand, low surface coverage at low pH is assigned to the highly positive charged surface.

Photocatalytic investigations revealed that coupling agents exhibiting covalent interaction with the titanium dioxide surface show degradation of the organic moiety, while the covalent connection between TiO_2_ and phosphate or carboxylate is stable under UV irradiation. Coupling agents which are adsorbed by electrostatic interaction on the other hand are desorbed from the TiO_2_ surface owing to the extensive formation of radicals which can combine with the ionic species present on the surface. Moreover, it was found that among the investigated coupling agents phosphonate exhibits the highest stability under photocatalytic conditions.

## Supporting Information

Supporting information includes XRD and DLS data, thermogravimetric analysis, and elemental analysis along with FTIR spectra above 2800 cm^−1^.
