# Supplementary material for: Interaction and UV-Stability of Various Organic Capping Agents on the Surface of Anatase Nanoparticles
Source: Materials (Basel). 2014 Apr 10;7(4):2890–912. doi: 10.3390/ma7042890 (PMC5453362; doi:10.3390/ma7042890)

Supporting Information

Synthesis of Dodecylphosphonic Acid

15.6 mL (64 mmol) of 1-bromododecane and 12.4 mL (74 mmol) of triethylphosphite were stirred for 3.5 h under refluxing conditions. The lower boiling products were removed by heating at 100 ℃ under ambient pressure conditions. The excess of triethyle phosphate was removed by distillation under reduced pressure. 13.1 g (42 mmol, 66% yield) of the intermediate product were obtained as a colorless liquid.

Spectroscopic Data of Intermediate-Product:

^{1}H NMR(CDCl3, δ, ppm): 0.85 (t, 3H, CH_2–CH_3); 1.22–1.33 (m, 24H, –CH_2–, CH_3–CH_2–O); 1.50–1.77 (m, 4H, CH_2–CH_2–P); 4.07 (m, 4H, CH_3–CH_2–O).

To 13.1 g (42 mmol) of the intermediate product (dodecylphosphonic acid, diethylester) 70 mL of concentrated HCl were added and the reaction mixture was heated to reflux for 22 h. The solvent and volatile byproducts were removed in vacuo. The crude product was washed with acetonitrile several times and dried under reduced pressure. After recrystallisation from n-hexane 9.5 g (90%) of colorless crystals were obtained.

Spectroscopic data for the product:

^{1}H NMR ($CDCl_3$, δ, ppm): 0.86 (t, 3H, CH_3); 1.20–1.43 (m, 18H, –CH_2–); 1.55–1.82 (m, 4H, CH_2–CH_2–P); 4.51 (s, 2H, P–OH);
^{13}C NMR ($CDCl_3$, δ, ppm): 14.2 (CH_2–CH_3); 21.9 (CH_2–CH_2–P); 22.8 (CH_2-CH_3); 24.0–29.6 (–CH_2–); 30.4 (–CH_2–P); 31.9 (CH_2–CH_2–CH_2–P);
^{31}P NMR ($CDCl_3$, δ, ppm): 38.0.

Figure S1. XRD of sol-gel synthesized titania nanoparticles.

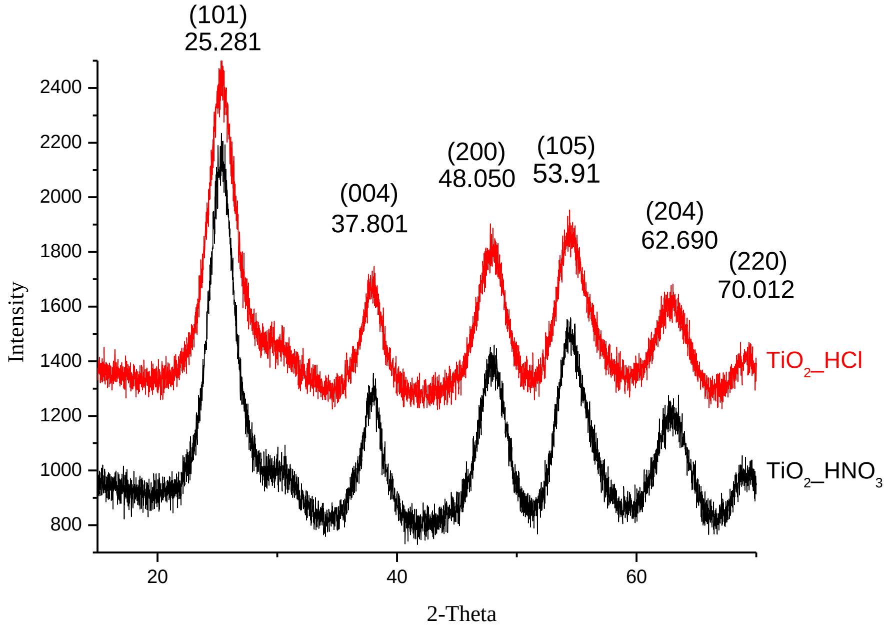

Figure S2. Dynamic light scattering (DLS) of titania nanoparticles.

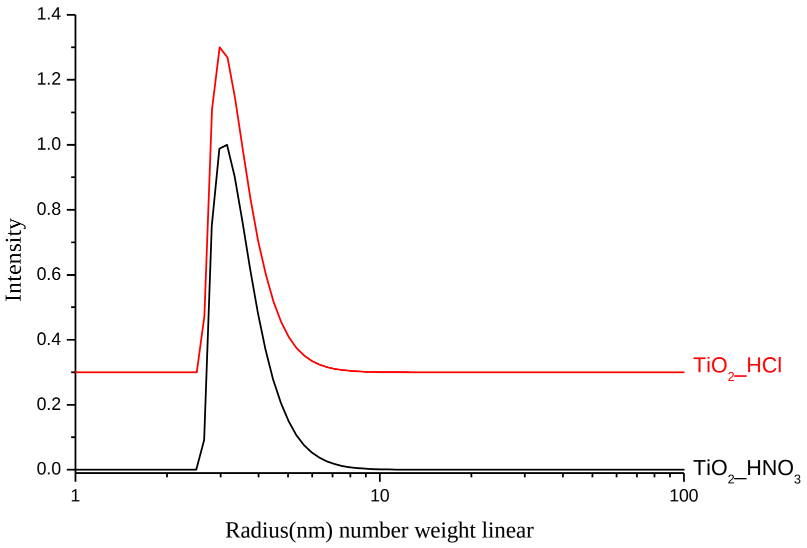

Figure S3. FT-IR of titania nanoparticles.

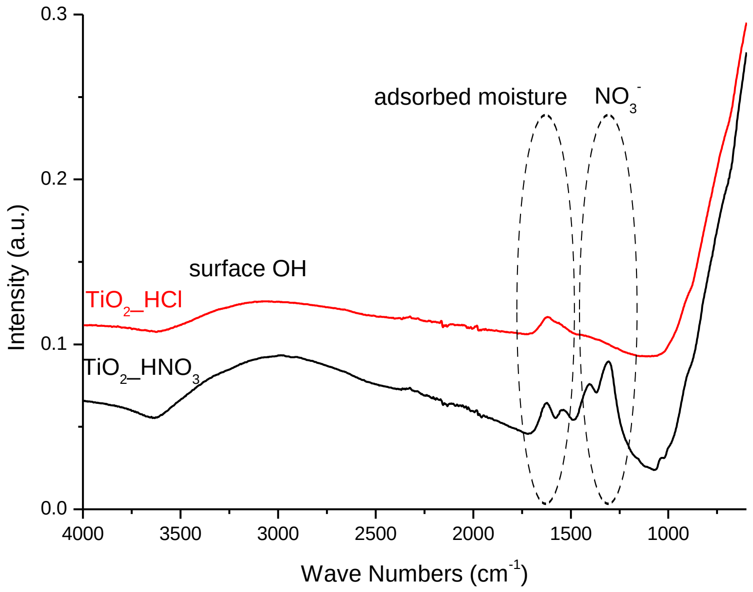

Figure S4. FT-IR (3200–2800 cm^{-1}) of SDS@TiO_2 at pH 1, 2, 3, 4 and 5.

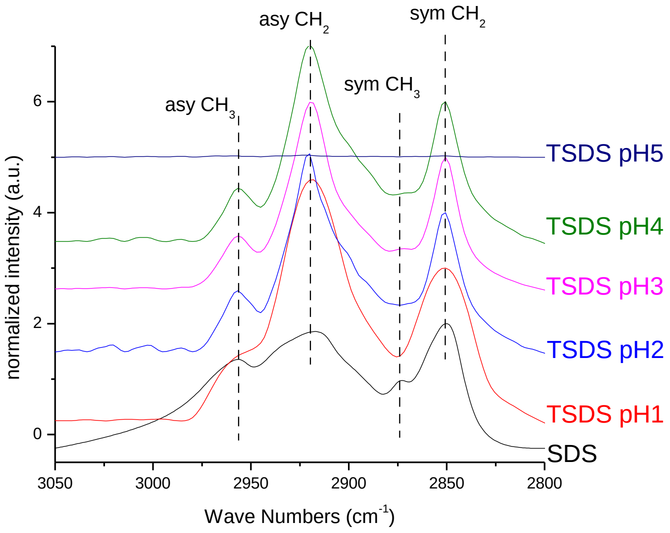

Figure S5. FT-IR (1800–600 cm^{-1}) of SDS@TiO_2 at pH 2, particles synthesized in HNO_3 or HCl respectively.

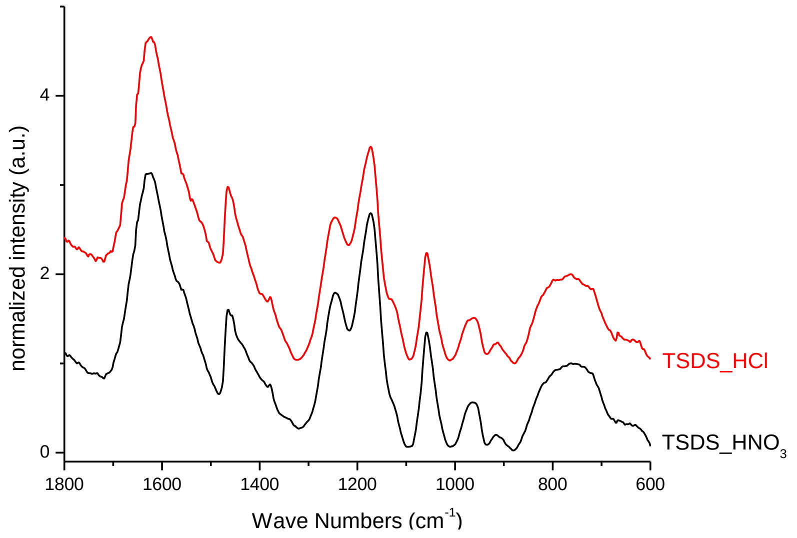

Figure S6. Thermograms of SDS@TiO_2 at pH 1, 2, 3, 4 and 5, particle synthesized in HNO_3.

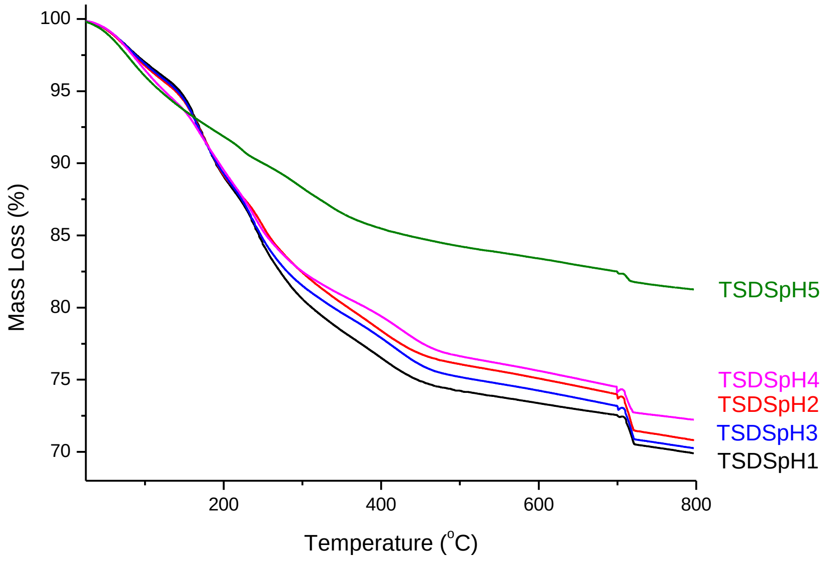

Figure S7. Thermograms of SDS@TiO_2 at pH 1, 2, 3, 4 and 5, particle synthesized in HCl.

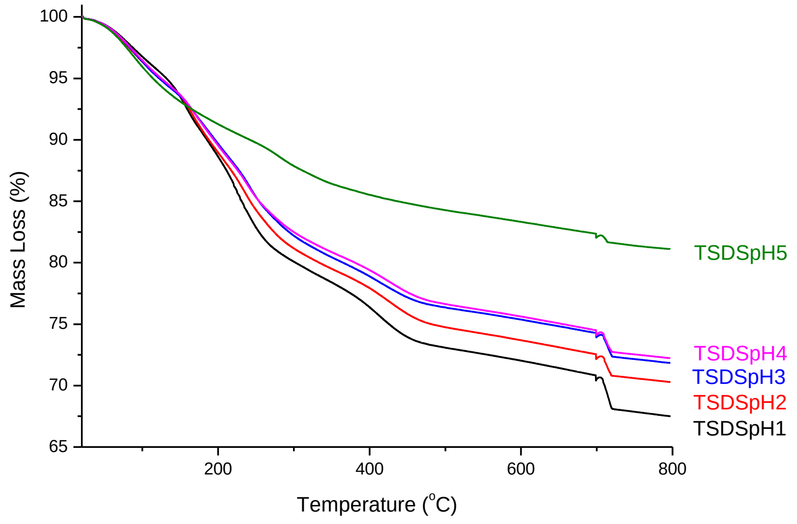

Figure S8. Thermograms of titania SDS@TiO_2 at pH 2 with different concentrations of SDS.

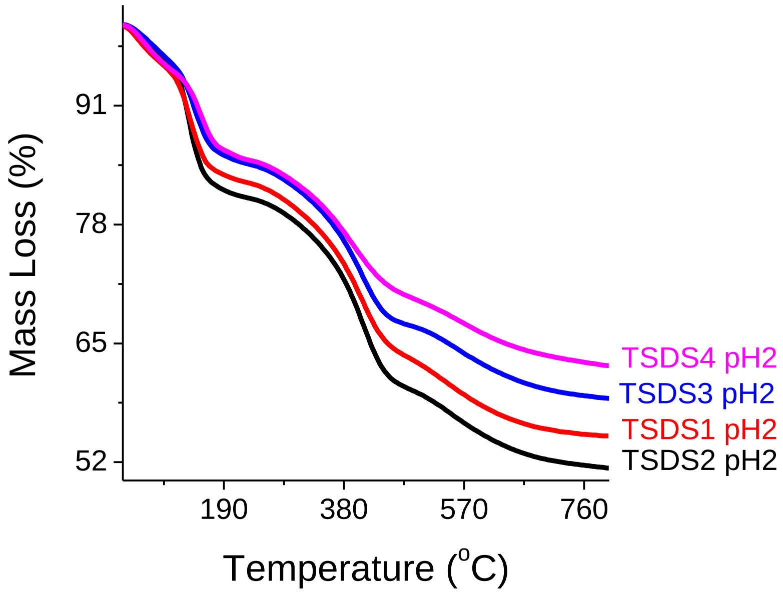

Table S1. Mass losses and surface coverage of SDS@TiO_2, particles synthesized in HNO_3.

pH	SDS Mass loss (%)	Surface coverage (Molecules/nm^2)
pH1	15.00	2.3
pH2	14.45	2.3
pH3	14.04	2.2
pH4	13.80	2.2
pH5	8.58	1.3

Table S2. Mass losses and surface coverage of SDS@TiO_2, particles synthesized in HCl.

pH	SDS Mass loss (%)	Surface coverage (Molecules/nm^2)
pH1	15.95	2.5
pH2	15.06	2.3
pH3	14.15	2.2
pH4	13.84	2.2
pH5	8.60	1.3

Table S3. Elemental Analyses of SDS@TiO_2 at pH 1, 2, 3, 4 and 5, particles synthesized in HNO_3.

No.	Sample ID	wt% C	wt% H	wt% N
1	SDS @ TiO_2 pH1	8.02	2.70	0.20
2	SDS @ TiO_2 pH2	7.48	1.98	0.23
3	SDS @ TiO_2 pH3	5.73	1.90	0.35
4	SDS @ TiO_2 pH4	9.14	2.40	0.21
5	SDS @ TiO_2 pH5	3.45	1.63	0

Table S4. Elemental Analyses of SDS@TiO_2 at pH 1, 2, 3, 4 and 5, particles synthesized in HCl.

No.	Sample ID	wt% C	wt% H	wt% N
1	SDS @ TiO_2 pH1	11.27	2.70	0
2	SDS @ TiO_2 pH2	9.52	2.47	0
3	SDS @ TiO_2 pH3	8.98	2.43	0
4	SDS @ TiO_2 pH4	8.98	2.41	0
5	SDS @ TiO_2 pH5	2.92	1.71	0.12

Figure S9. FT-IR spectra (3050–2800 cm^{-1}) of DDA@TiO_2 at pH 1, 2, 3, 4 and 5.

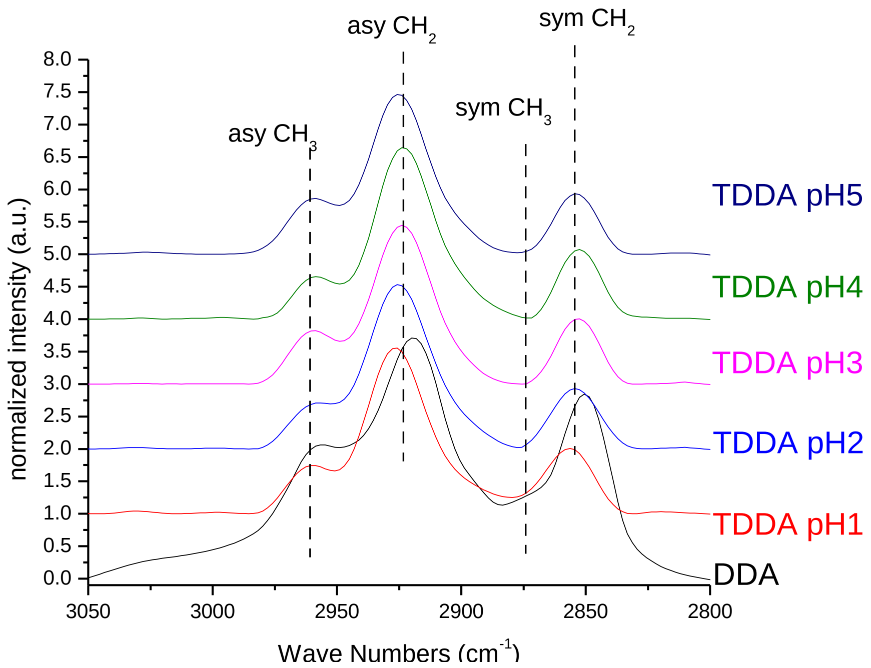

Figure S10. TGA of DDA@TiO_2 at pH 1, 2, 3, 4 and 5.

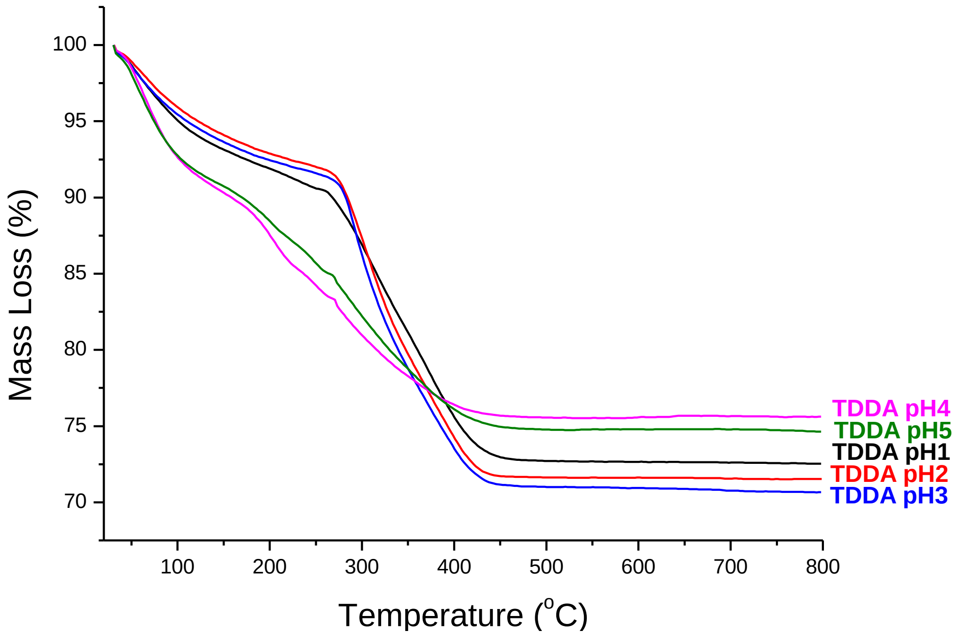

Figure S11. TGA of DDA@TiO_2 at pH 3 with different concentrations of DDA.

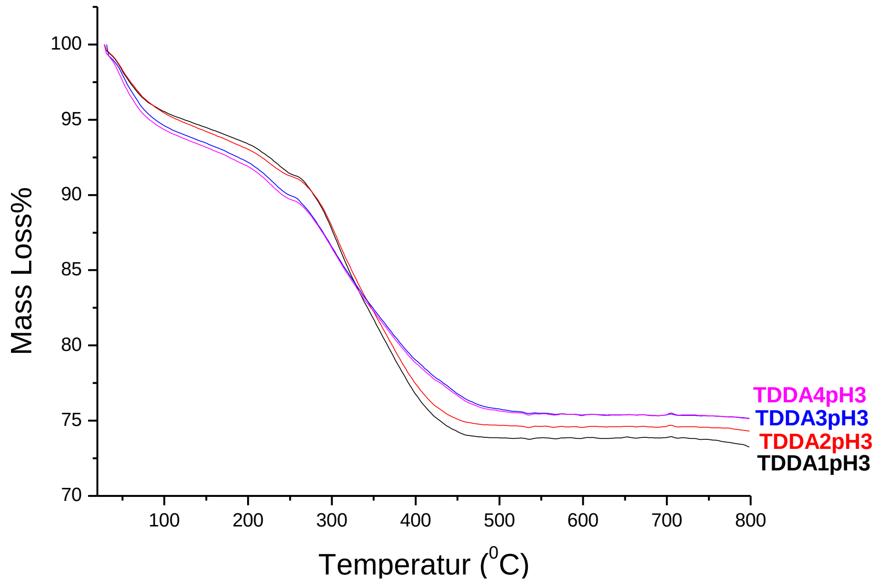

Table S5. Mass losses and surface coverage of DDA@TiO_2.

pH	DDA Mass loss (%)	Surface coverage (Molecules/nm^2)
pH 1	18.62	3.7
pH 2	20.04	4.0
pH 3	21.1	4.1
pH 4	10.25	2.0
pH 5	12.53	2.5

Figure S12. TGA of DDAmine@TiO_2 at pH 1, 2, 3, 4 and 5**.**

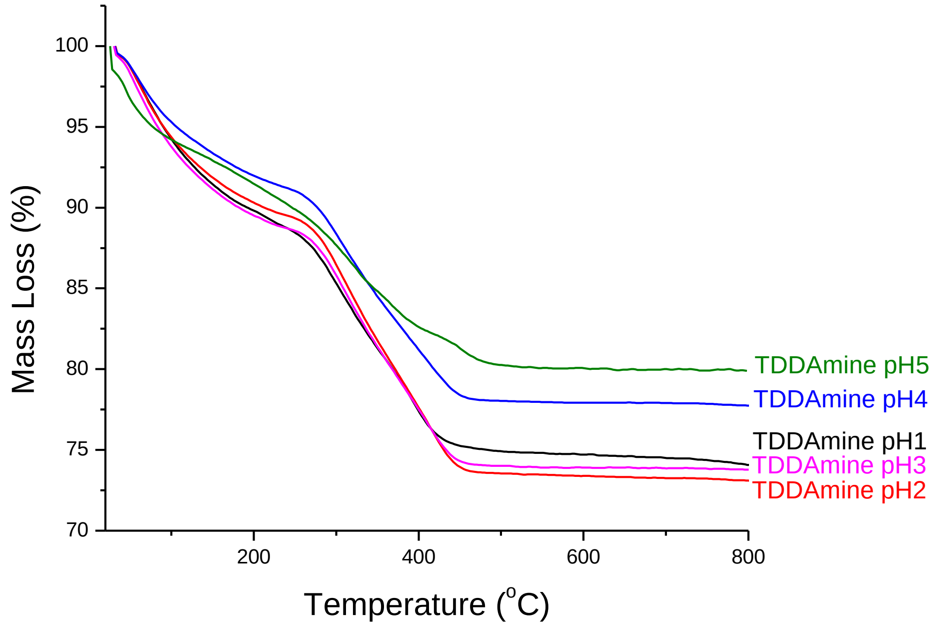

Table S6. Elemental analysis of DDAmine@TiO_2 at pH 1, 2, 3, 4 and 5.

Sample	wt% C	wt% H	wt% N
TDDAmine pH1	11.10	2.50	0.95
TDDAmine pH2	12.13	2.91	1.18
TDDAmine pH3	11.52	2.32	0.89
TDDAmine pH4	10.6	2.40	1.09
TDDAmine pH5	7.33	1.81	0.81

Table S7. Mass losses and surface coverage of DDAmine@TiO_2.

pH	DDAmine Mass loss (%)	Surface coverage (Molecules/nm^2)
pH 1	14.54	3.3
pH 2	16.37	3.7
pH 3	15.2	3.4
pH 4	13.57	3.1
pH 5	10.13	2.3

Figure S13. TGA of DDAmine@TiO_2 at pH 2 with different concentrations of DDAmine.

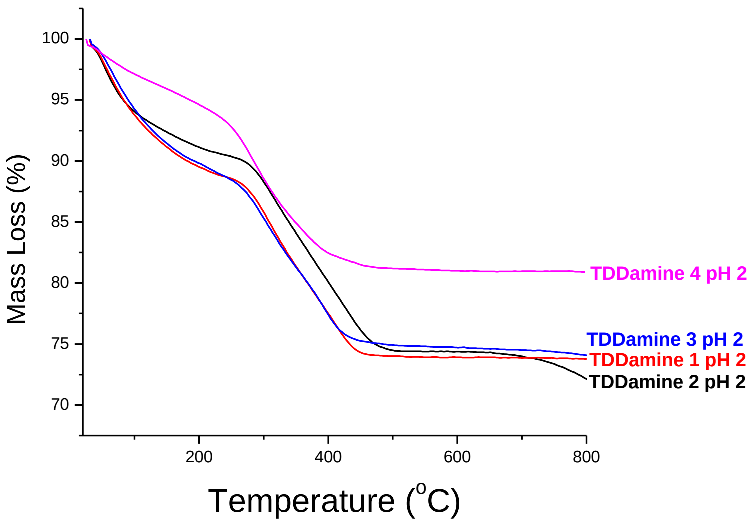

Figure S14. FT-IR spectra of DPA@TiO_2 at pH 1, 2, 3, 4 and 5.

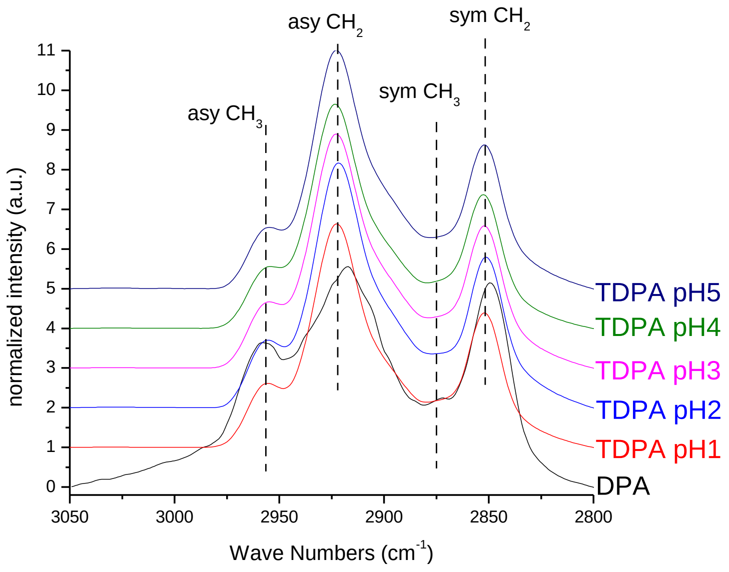

Figure S15. TGA of DPA@TiO_2 at pH1to pH5.

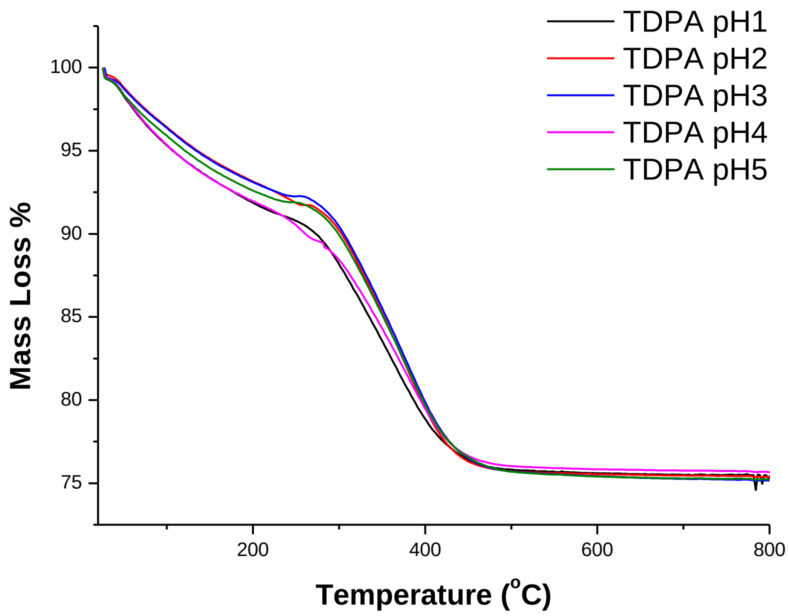

Figure S16. Thermograms of DPA@TiO_2 with different concentrations of DPA at pH3.

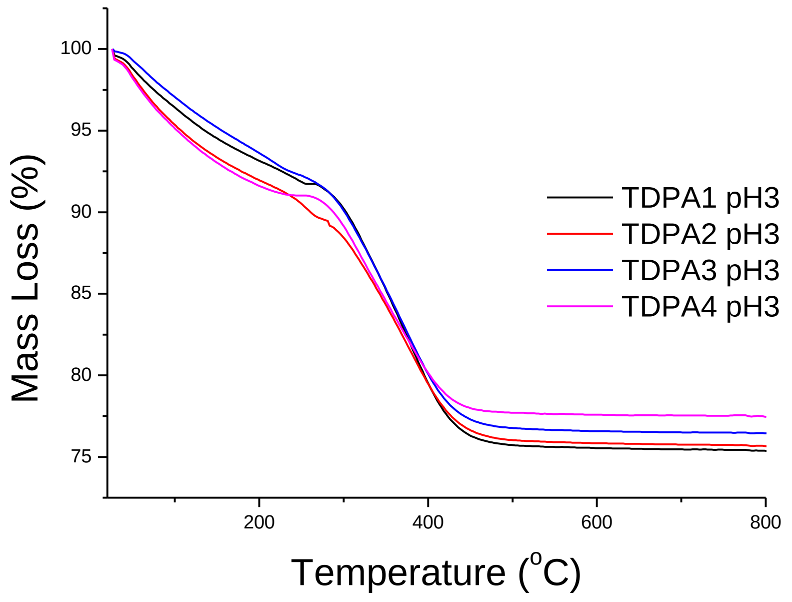

Table S6. Elemental analyses of DPA@TiO_2 at pH = 1 to pH = 5.

Sample	wt% C	wt% H	wt% P
TDPA pH 1	9.28	1.72	0.849
TDPA pH 2	11.13	2.85	1.03
TDPA pH 3	11.58	2.91	1.18
TDPA pH 4	9.59	2.32	1.08
TDPA pH 5	8.33	2.01	0.90

Table S7. Mass loss and surface coverage of DPA@TiO_2.

pH	DPA Mass loss (%)	Surface coverage (Molecules/nm^2)
pH 1	15.5	3.8
pH 2	16.2	4.0
pH 3	16.7	4.1
pH 4	15.3	3.7
pH 5	14.7	3.6

Figure S17. FT-IR spectra (KBr: 2 mg sample, 150 mg KBr) of SDS@TiO_2 after different times of illumination, particles synthesized in HNO_3.

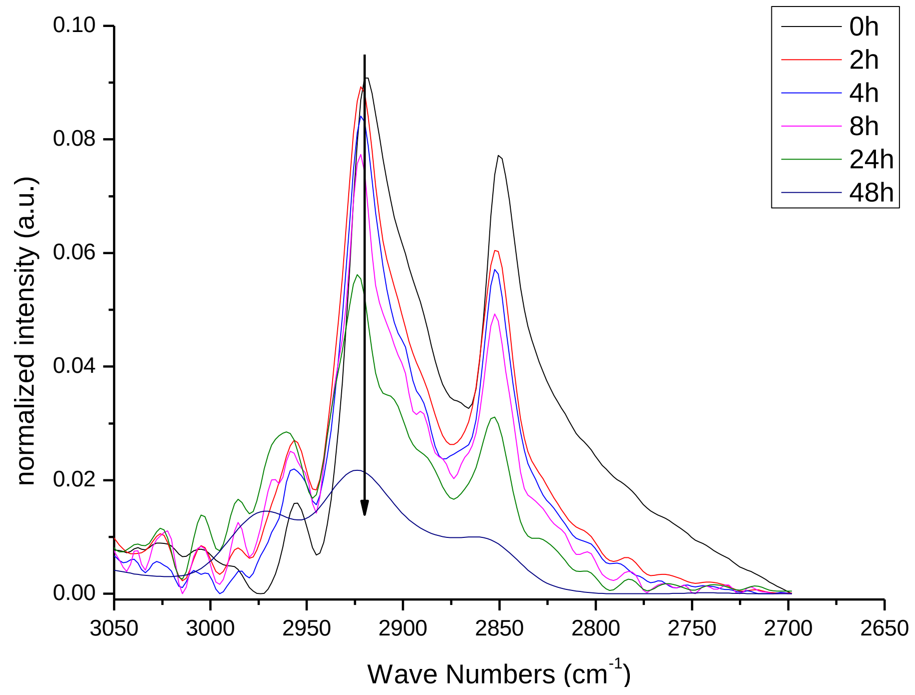

Figure S18. FT-IR spectra (KBr: 2 mg sample, 150 mg KBr) of SDS@TiO_2 after different times of illumination, particles synthesized in HCl.

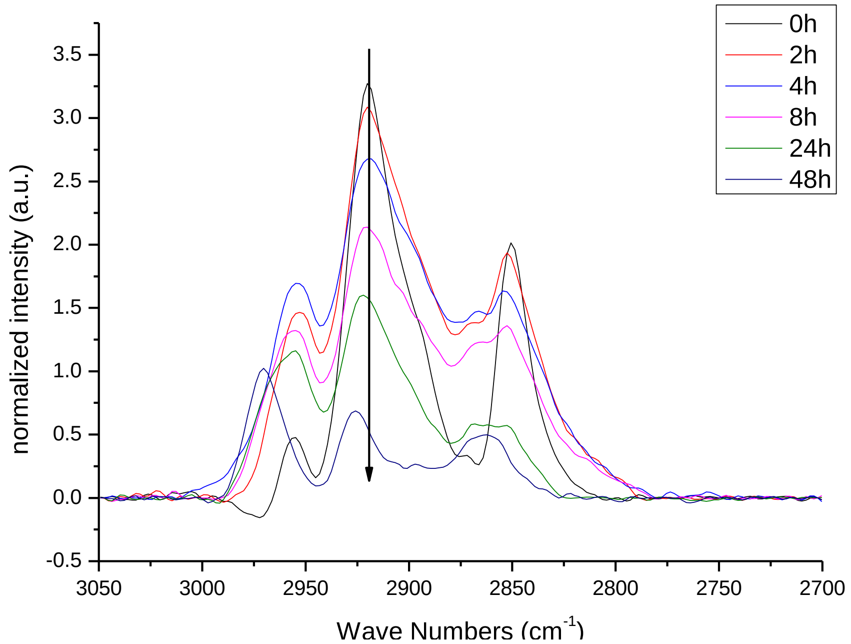

Figure S19. Kinetic plot for determining the first-order reaction rate constant k of $SDS@TiO_2$.

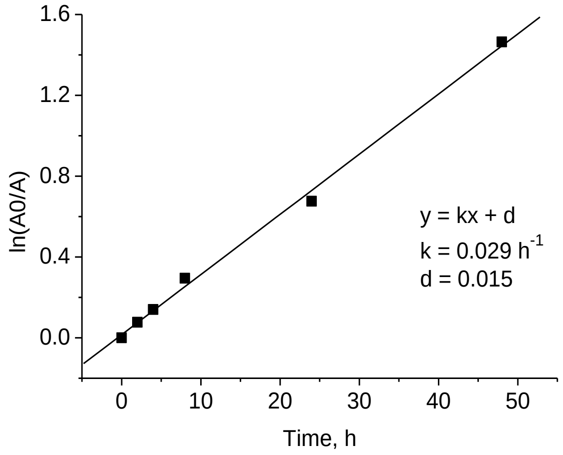

Figure S20. FT-IR spectra (KBr: 2 mg sample, 150 mg KBr) of $DDA@TiO_2$ after different times of illumination.

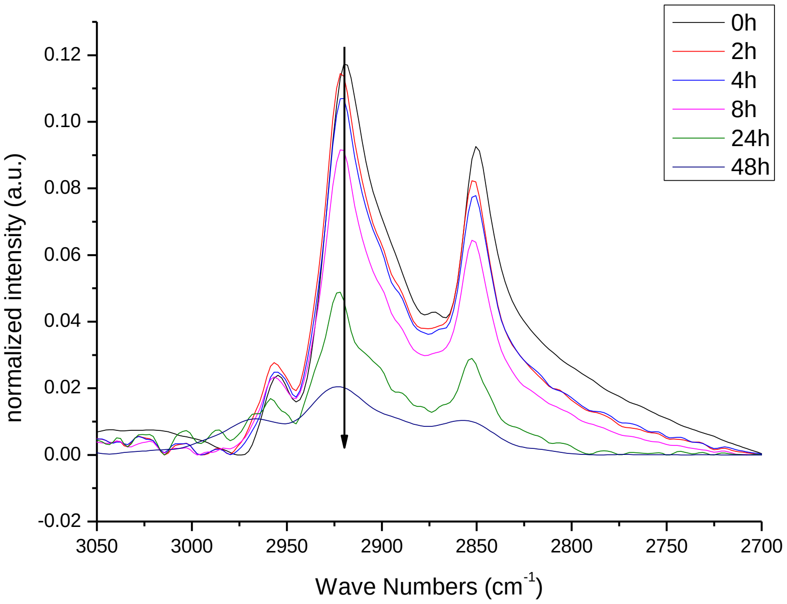

Figure S21. Kinetic plot for determining the first-order reaction rate constant k of $DDA@TiO_2$.

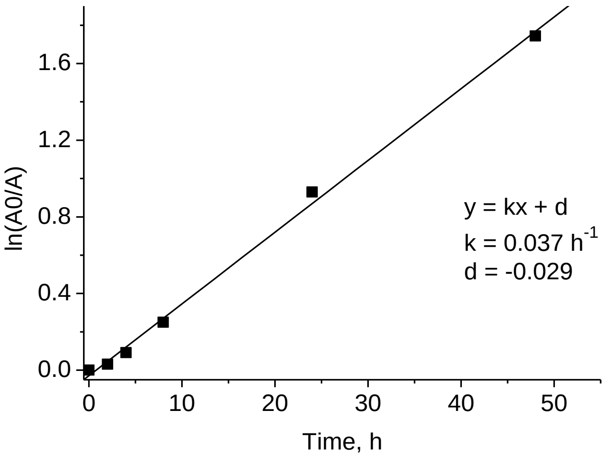

Figure S22. FT-IR spectra (KBr: 2 mg sample, 150 mg KBr) of DDAmine@TiO_2 after different times of illumination.

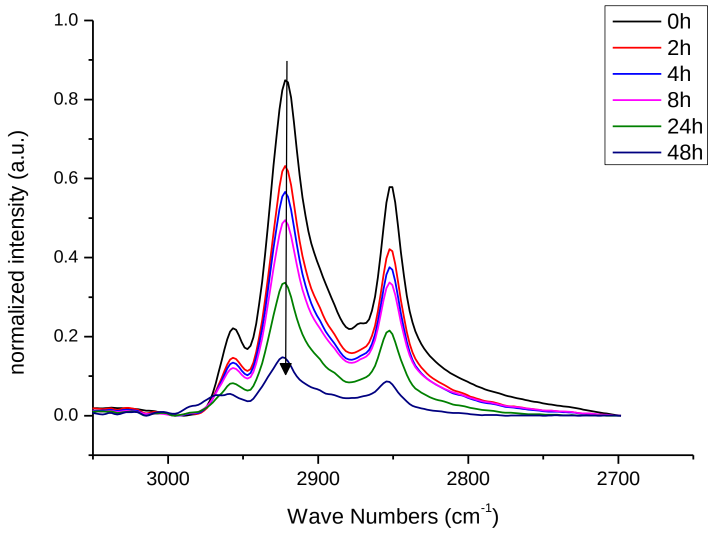

Figure S23. Kinetic plot for determining the first-order reaction rate constant k of DDAmine@TiO_2.

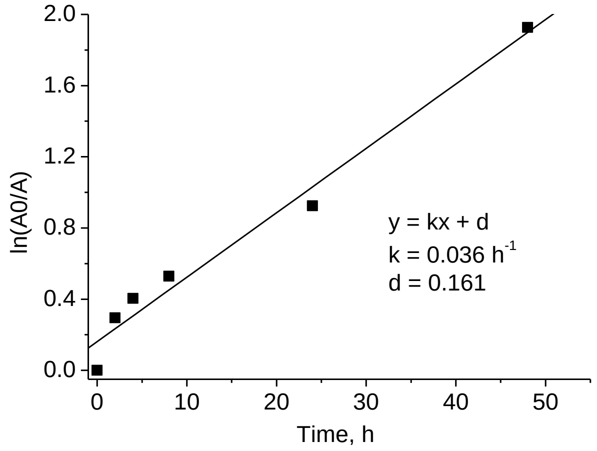

Figure S24. FT-IR spectra (KBr: 2 mg sample, 150 mg KBr) of DPA@TiO_2 after different times of illumination.

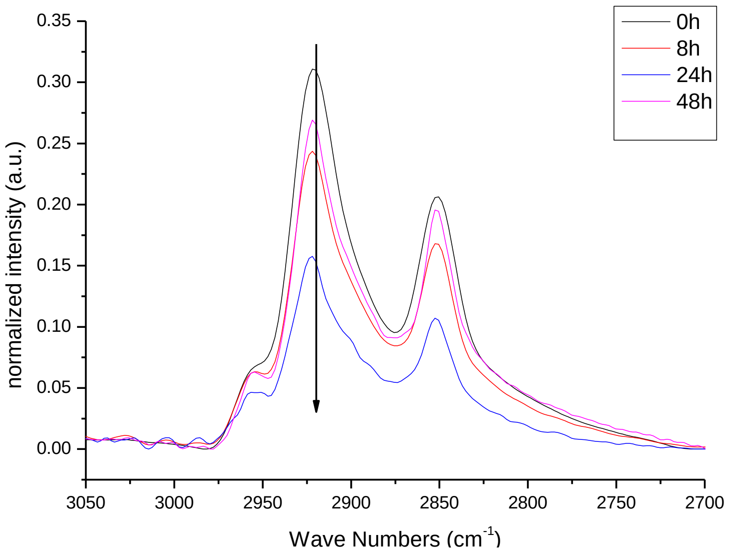

Figure S25. Kinetic plot for determining the first-order reaction rate constant k of DPA@TiO_2.

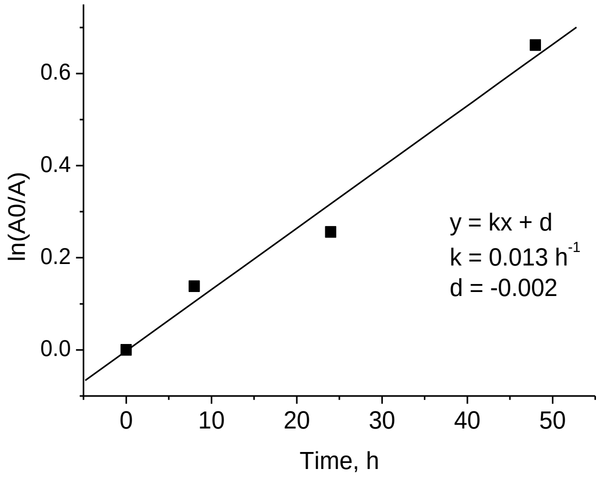

Figure S26. ^{31}P MAS NMR spectra of DPA@TiO_2 without illumination and after 16 days of illumination.

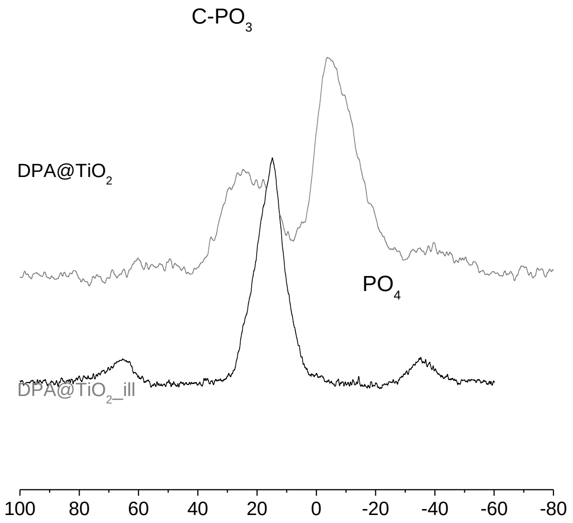

Figure S27. FT-IR spectra (KBr: 2 mg sample, 150 mg KBr) of SDS@TiO_2 after different times of illumination.

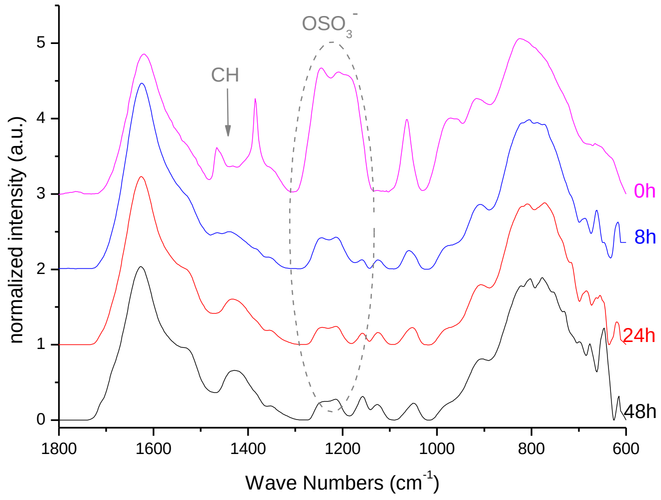

Figure S28. FT-IR spectra (KBr: 2 mg sample, 150 mg KBr) of DDAmine@TiO_2 after different times of illumination.

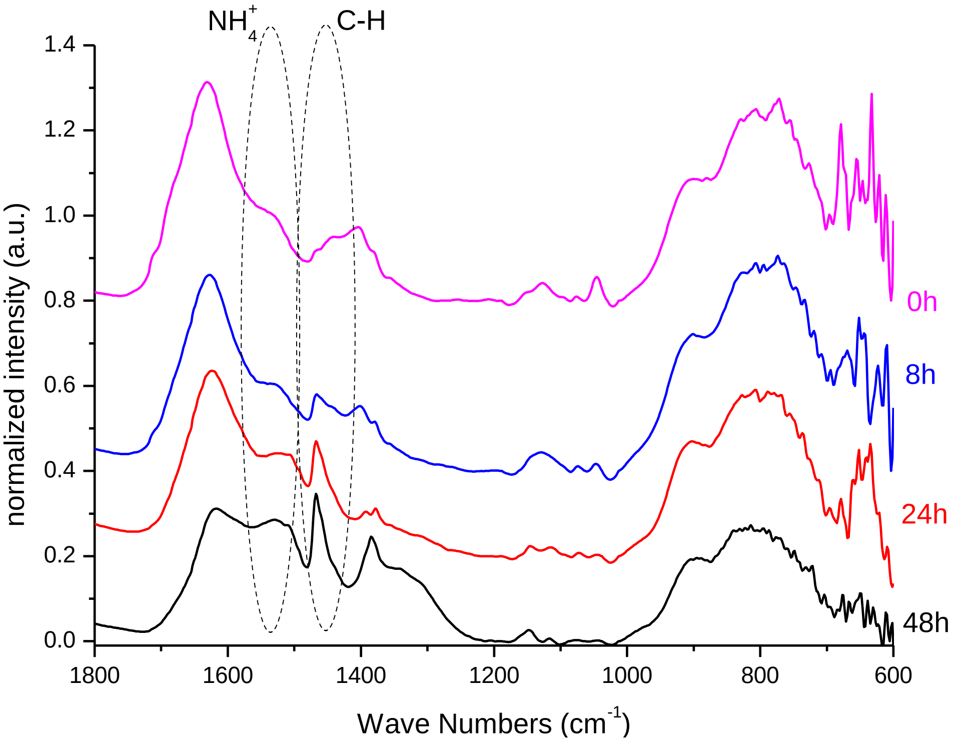

Figure S29. FT-IR spectra (1600–700 cm^{-1}) of SDS@TiO_2 functionalized at pH 1, 2, 3, 4 and 5 in HCl.

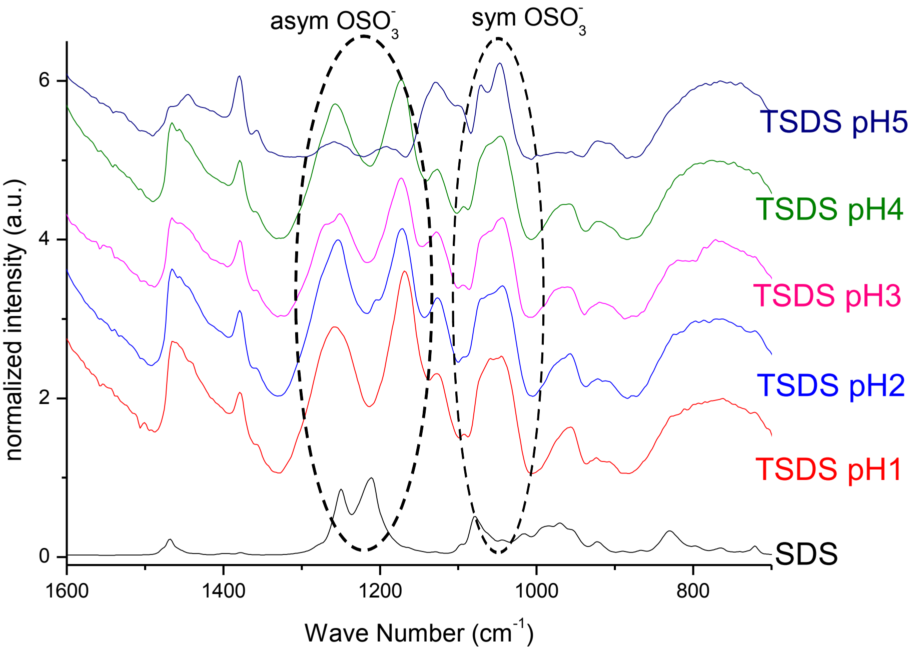

Supplement: Supplementary file 1 [file materials-07-02890-s001.pdf]
